# Multicentre open label randomised controlled trial of immediate enhanced ambulatory ECG monitoring versus standard monitoring in acute unexplained syncope patients: the ASPIRED study

**DOI:** 10.1136/bmjopen-2022-069530

**Published:** 2023-02-23

**Authors:** Matthew J Reed, Neil Grubb, Chris Lang, Steve Goodacre, Rachel O’Brien, Christopher J Weir, Praveen Thokala, Nicola Freeman, Caroline Blackstock, Lynn Dinsmore, Julia Boyd, Imad Adamestam, Pam Macrae, Robert Hannigan, Trudie Lobban

**Affiliations:** 1 Emergency Medicine Research Group Edinburgh (EMERGE), Royal Infirmary of Edinburgh, Edinburgh, UK; 2 Acute Care Edinburgh, Usher Institute, University of Edinburgh, Edinburgh, UK; 3 Department of Cardiology, Royal Infirmary Edinburgh, Edinburgh, UK; 4 School of Health and Related Research (ScHARR), University of Sheffield, Sheffield, UK; 5 Edinburgh Clinical Trials Unit, Usher Institute, University of Edinburgh, Edinburgh, UK; 6 ASPIRED study Patient Advisory Group, Edinburgh, UK; 7 Arrhythmia Alliance, Chipping Norton, UK

**Keywords:** ACCIDENT & EMERGENCY MEDICINE, Adult cardiology, Pacing & electrophysiology, GENERAL MEDICINE (see Internal Medicine), Cardiology

## Abstract

**Introduction:**

Diagnosing underlying arrhythmia in emergency department (ED) syncope patients is difficult. There is a evidence that diagnostic yield for detecting underlying arrhythmia is highest when cardiac monitoring devices are applied early, ideally at the index visit. This strategy has the potential to change current syncope management from low diagnostic yield Holter to higher yield ambulatory monitoring, reduce episodes of syncope, reduce risk of recurrence and its potential serious consequences, reduce hospital admissions, reduce overall health costs and increase quality of life by allowing earlier diagnosis, treatment and exclusion of clinically important arrhythmias.

**Methods and analyses:**

This is a UK open prospective parallel group multicentre randomised controlled trial of an immediate 14-day ambulatory patch heart monitor vs standard care in 2234 patients presenting acutely with unexplained syncope. Our patient focused primary endpoint will be number of episodes of syncope at 1 year. Health economic evaluation will estimate the incremental cost per syncope episode avoided and quality-adjusted life year gained.

**Ethics and dissemination:**

Informed consent for participation will be sought. The ASPIRED trial received a favourable ethical opinion from South East Scotland Research Ethics Committee 01 (21/SS/0073). Results will be disseminated via scientific publication, lay summary and visual abstract.

**Trial registration number:**

ISRCTN 10278811.

**STRENGTHS AND LIMITATIONS OF THIS STUDY**

- The ASPIRED trial uses non-blinded randomisation to compare immediate enhanced ambulatory ECG monitoring versus standard monitoring in acute unexplained syncope patients.
- The primary outcome measure is a patient focussed measure very relevant to the population of patients who suffer syncope.
- We are using health economic analysis and a separate embedded qualitative study to assess the wider implications of using immediate enhanced ambulatory ECG monitoring in this population.
- In contrast to the few published studies on ambulatory monitoring in syncope patients which are generally single centred, without any comparative group and with heterogeneous patient populations and device capabilities, this study is multicentred and includes a standard care group.

## Introduction

### Background and rationale

Syncope (or blackout) is common; 650 000 patients present to UK emergency departments (EDs) every year. The three underlying causes are neurocardiogenic (including simple faint), postural hypotension (blood pressure fall on standing) and cardiac disease (structural heart disease or cardiac arrhythmia). Diagnosis is difficult and is not made in around 50% of patients after assessment.^1–4^ While vasovagal and postural syncope are relatively benign, serious pathology (affecting 7 out of every 100 patients at 1 month after ED attendance^5^) include arrhythmia (an abnormal heart rhythm). When cardiac arrhythmias are detected, they are most commonly asystolic pauses, reflex bradycardia or advanced atrioventricular block, with tachycardia being the minority.^6^

The difficulty in the ED is to differentiate between the causes of syncope and identify patients at higher risk. This can be complicated as many patients have fully recovered on ED arrival and their examination and presenting ECG may both be normal. The current method for establishing cardiac arrhythmia as the cause of syncope rests on correlating the arrhythmia with symptoms. The lack of efficacy and availability of commonly used monitoring devices means most high and medium risk patients are admitted to the hospital for observation and telemetry (if available), with escalating costs.^1^ Unfortunately, many (around 50% of patients after assessment) still end up being discharged without a diagnosis.^7^

In general, syncope reoccurs in around 50% of patients within a year. Recurring episodes increase hospital admissions, health costs and importantly reduce the quality of life of patients. While there is a wide variation in the literature with respect to the number of syncope episodes and recurrence rates pretreatment and post-treatment^6 8–10^ once a cardiac arrhythmia diagnosis is made and treatment initiated, around only 10% of patients will have a 1-year recurrence^11^ and syncope episodes will drop by over 90%.^11^

There is evidence that the diagnostic yield for detecting underlying arrhythmia is highest when cardiac monitoring devices are applied early after syncope, ideally at the index visit^1–3^. To solve the problems with current routine ECG monitoring devices, several novel ambulatory devices have recently been developed^1 12–15^. These devices are non-invasive, water-resistant, have no leads or wires, are discreet to wear and are CE-marked for clinical use in the UK. They continuously monitor the heart for up to 14 days including during sleep, in the shower and during moderate exercise and some have a button for patients to capture symptomatic events. They offer medium duration high fidelity ECG recording and are well tolerated^16 17^. At the end of the monitoring period, patients return the devices back to the company (by email) or NHS (to be downloaded) for analysis. The PATCH-ED pilot study helped establish trial methods and number of available participants informing this randomised controlled trial (RCT)^1^. This pilot study showed a novel ECG monitoring device was able to detect serious cardiac arrhythmia requiring treatment in a significant proportion of patients and demonstrated the potential to influence clinical management decisions relating to hospital admission and participant outcomes.

The ASPIRED RCT will compare a novel ambulatory cardiac monitoring device with standard practice in syncope patients. We hypothesise that applying cardiac monitoring early after syncope at the index visit is the optimum strategy to detect, diagnose, treat and exclude underlying cardiac arrhythmia.

### Study objectives

#### Primary

To determine whether immediate, enhanced (14 days) ambulatory ECG monitoring decreases the number of self-reported episodes of syncope at 1 year compared with standard care monitoring in acute unexplained syncope patients.

#### Secondary

To determine whether immediate, enhanced (14 days) ambulatory ECG monitoring in acute unexplained syncope patients can:

1. Decrease the number of episodes of self-reported syncope at 90 days, and 2 years compared with standard care monitoring.
2. Decrease the time to detection of clinically significant cardiac arrhythmia compared with standard care monitoring.
3. Increase the rate of detection of clinically significant cardiac arrhythmia at 90 days and 1 year compared with standard care monitoring.
4. Increase the rate of ECG/symptom correlation at 90 days and 1 year compared with standard care monitoring.
5. Demonstrate cost-effectiveness compared with standard care monitoring.
6. Decrease the number of episodes of syncope identified in the medical records at 90 days, 1 and 2 years compared with standard care monitoring.
7. Decrease the index hospital admission rate and duration of hospital stay compared with standard care monitoring.
8. Decrease 90 days, 1-year and 2-year syncope recurrence rates (identified in the medical records and self-reported) compared with standard care monitoring.
9. Increase patient satisfaction compared with standard care monitoring.
10. Decrease the rate of 30 day, 1-year and 2-year all-cause death compared with standard care monitoring.
11. In the intervention group, by reporting the timing of detection of clinically significant cardiac arrhythmia what is the optimum duration of acute ambulatory ECG monitoring.
12. Increase the affect rate of diagnostic testing and therapeutic intervention.

## Methods and analysis

### Study design

This is a UK open prospective parallel group multicentre RCT of an immediate 14-day ambulatory patch heart monitor versus standard care monitoring in 2234 participants presenting acutely with unexplained syncope. The patient focused primary endpoint will be number of episodes of syncope at 1 year (table 1).

**Table 1:** Study summary in PICO format

P: Population	Adults presenting acutely to UK hospitals with syncope remaining unexplained after initial ED/AMU assessment
I: Intervention	14-day ambulatory heart monitor placed on patients
C: Comparator	Standard care monitoring
O: Primary Outcome	Number of self-reported episodes of syncope at 1 year

AMU, acute medicine unit; ED, emergency department.

#### Study eligibility

Two thousand two hundred and thirty-four adult (16 years or older) participants presenting acutely to UK hospitals with syncope remaining unexplained after initial ED or acute medicine unit (AMU) assessment will be enrolled. Syncope will be defined as transient loss of consciousness (TLOC) with inability to maintain postural tone and immediate complete spontaneous recovery without medical intervention^18^.

##### Inclusion criteria

1. Syncope remains unexplained after initial ED/AMU assessment.
2. Aged ≥16 years.
3. Patient has capacity.
4. Local resident (ie, resident within local health board so will not be lost to medical record follow-up).
5. <5 self-reported episodes of syncope in the previous month.

##### Exclusion criteria

1. Obvious underlying cause after assessment.
  - Features of vasovagal syncope (see box 1) AND absence of structural heart disease AND normal physical examination AND normal ECG.
  - Arrhythmia on prehospital or hospital ECG as likely cause of syncope.
  - Postural hypotension (symptomatic postural drop >20 mm Hg AND suggestive history).
  - Confirmed diagnosis of pulmonary embolus or acute myocardial infarction.
  - Radiological diagnosis or clinical signs/symptoms of cerebrovascular accident/transient ischaemic attack or subarachnoid haemorrhage.
  - Evidence of:
    - Haemorrhage.
    - Alcohol or illicit drugs.
    - Epileptic seizure.
    - Hypoglycaemia.
    - Head trauma.
    - Other obvious cause of syncope as presumptive cause of TLOC.
2. Inability to consent.
3. Previous recruitment into the study.
4. Patient in custody or prison.
5. Aged<16 years.
6. Patient does not reside within local health board and will therefore be lost to medical record follow-up.
7. Five or more self-reported episodes of syncope in previous 4 weeks.

Pregnancy is not an exclusion criteria.

Box 1 **Features of vasovagal/postural syncope**

Associated with typical symptoms of reflex syncope (eg, light-headedness, feeling of warmth, nausea, vomiting).

After sudden unexpected unpleasant sight, sound, smell or pain.

In association with micturition, defaecation, cough, laughter, venepuncture, blood phobia.

After prolonged standing or crowded, hot places.

During a meal or after eating a meal.

With head rotation or pressure on carotid sinus (eg, tumour, shaving, tight collars).

Associated with standing up quickly from a sitting or lying position.

Long history (years) of recurrent syncope with low-risk features with the same characteristics of the current episode.

#### Participant recruitment

**Figure 1:**
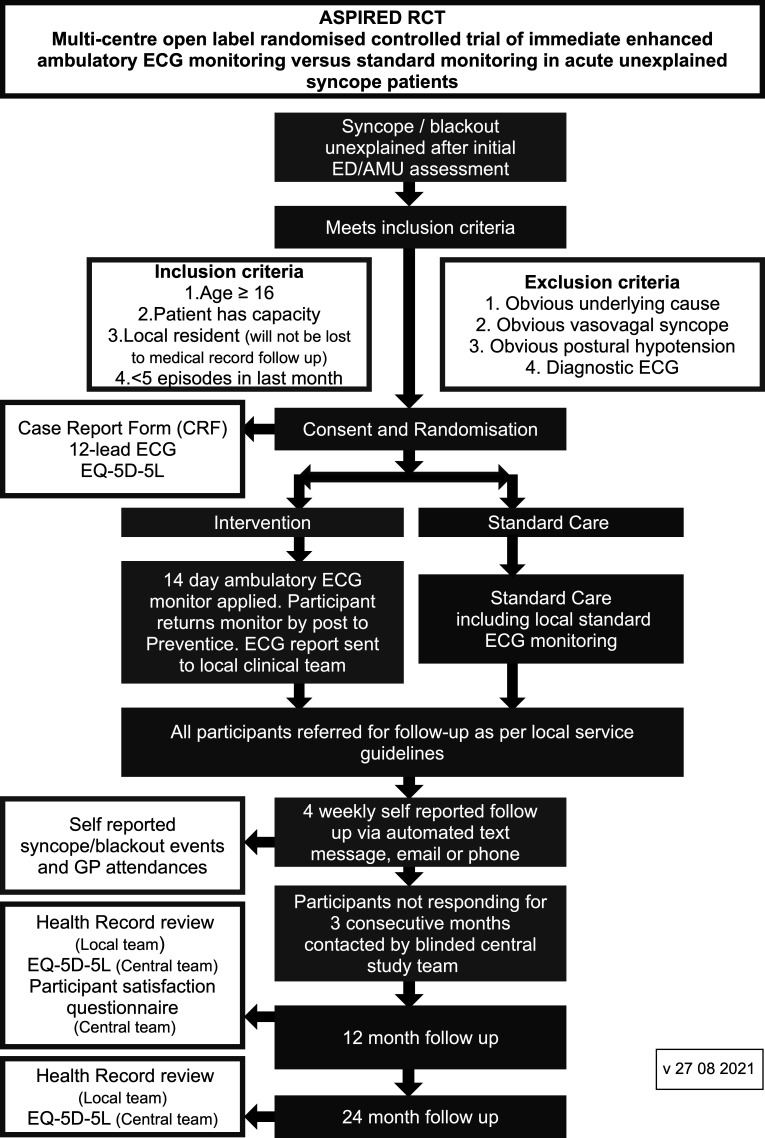
Schematic diagram of the study design and participant recruitment. AMU, acute medicine unit; ED, emergency department; GP, general practitioner.

Recruitment will take place in around 30–40 NHS acute tertiary and district hospitals. Figure 1 summarises the study design and participant recruitment.

### Randomisation and interventions

Randomisation will be performed using a web-based randomisation service to ensure allocation concealment, managed by Edinburgh Clinical Trials Unit (ECTU). The allocation sequence will be created by an ECTU database programmer using computer-generated pseudo-random numbers. Participants will be randomised, 1:1, between the two study arms. Stratification by site will be used to ensure there will not be a significant imbalance between the number randomised to intervention and control at any site. Stratification by other site-level characteristics will not be performed.

Standard care will include all care usually given to unexplained syncope patients at each participating site along with some form of standard care monitoring such as but not limited to wired inpatient telemetry, Holter style monitoring or implantable ECG recorder. The study will be conducted over 4 years. Recruitment will take place over 18 months. Intervention group participants will be fitted with a 14-day ambulatory heart monitor. All participants will be followed up for 2 years after index event.

#### Outcome measures

##### Primary endpoint

Number of self-reported episodes of syncope at 1 year.

##### Secondary endpoints

Within trial cost-effectiveness (cost per syncope avoided and cost per quality-adjusted life year (QALY) gained), and lifetime cost per QALY gained at (a) 1 year and (b) 2 years.
Number of self-reported episodes of syncope at (a) 90 days and (b) 2 years, those identified in the medical records at (c) 90 days, (d) 1 year and (e) 2 years, and syncope recurrence rate at (f) 90 days, (g) 1 year and (h) 2 years.
Index presentation hospital (a) admission rate and (b) duration of hospital stay.
Patient satisfaction (measured using a patient questionnaire) at 1 year.
Clinically significant cardiac arrhythmia (serious and/or symptomatic cardiac arrhythmia, box 2) at (a) 90 days, (b) 1 year and (c) 2 years.
(a) 30-day, (b) 1-year and (c) 2-year all-cause death.
Detection of diagnostic ECG/symptom correlation (symptomatic) at (a) 90 days, (b) 1 year and (c) 2 years.
Time to detect clinically significant cardiac arrhythmia (ie, time to clinician being aware).
In the intervention group, duration of enhanced ambulatory ECG monitoring required to detect clinically significant cardiac arrhythmia.
Number and type of diagnostic tests and therapeutic interventions at (a) 1 year and (b) 2 years.

Box 2 **Definitions of clinically significant cardiac arrhythmias**

Ventricular fibrillation.^*^

Ventricular tachycardia (VT) ≥120 beats per minute (bpm) for ≥30s.^*^

VT ≥120 bpm for <30 s (≥4 beats).^*^

Complete or 3rd degree heart block.^*^

Second degree atrioventricular heart block Mobitz type II.^*^

Second degree atrioventricular heart block Mobitz type I.

Pause ≥6 s.^*^

Sinus pause ≥2.5 s when awake or ≥4 s at night (but <6 s).

Sinus bradycardia <30 bpm.^*^

Bradycardia <40 bpm for ≥30 s.^*^

Bradycardia <40 bpm for <30 s.

Sick sinus syndrome with alternating sinus bradycardia and tachycardia.

Junctional/idioventricular rhythm ≥30 s in duration.

Supraventricular tachycardia >100 bpm ≥30 s in duration.

Atrial flutter/fibrillation with ventricular rate >100 bpm or <60 bpm ≥30 s in duration.

New atrial flutter/fibrillation ≥30 s in duration.

All arrhythmias will also be classed as symptomatic or asymptomatic during monitoring period.

*‘Serious’ clinically significant cardiac arrhythmia.

### Trial assessments

#### Participant identification

Potential participants will be identified from EDs/AMUs or other acute settings and approached either during their ED/AMU or hospital stay or contacted after discharge and invited to take part in the study.

#### Participant consent

Potential participants will be given a Participant Information Sheet (PIS). Potential participants will be randomised within 72 hours of their hospital attendance. If a patient has been discharged from the hospital, then they will be contacted by the local clinical team or research team (if part of the clinical team), and a copy of the PIS will be emailed or posted out to the patient. The PIS will also be available on the trial website. A delegated member of the local study team will seek verbal consent over the telephone and will sign the consent form on behalf of the participant. The original consent form will be filed in the Investigator Site File and a copy will be sent to the participant. Alternatively, the patient can attend the hospital with travel expenses, to provide written informed consent in person. The ambulatory ECG device can be sent to the participant’s address or collected from the local study team if randomised to the intervention arm. Each participating centre will upload screening information of non-identifiable potentially eligible patients who were approached to participate in the study, onto the study database. Participants are free to withdraw from the study at any point or can be withdrawn by the investigator.

**Figure 2:**
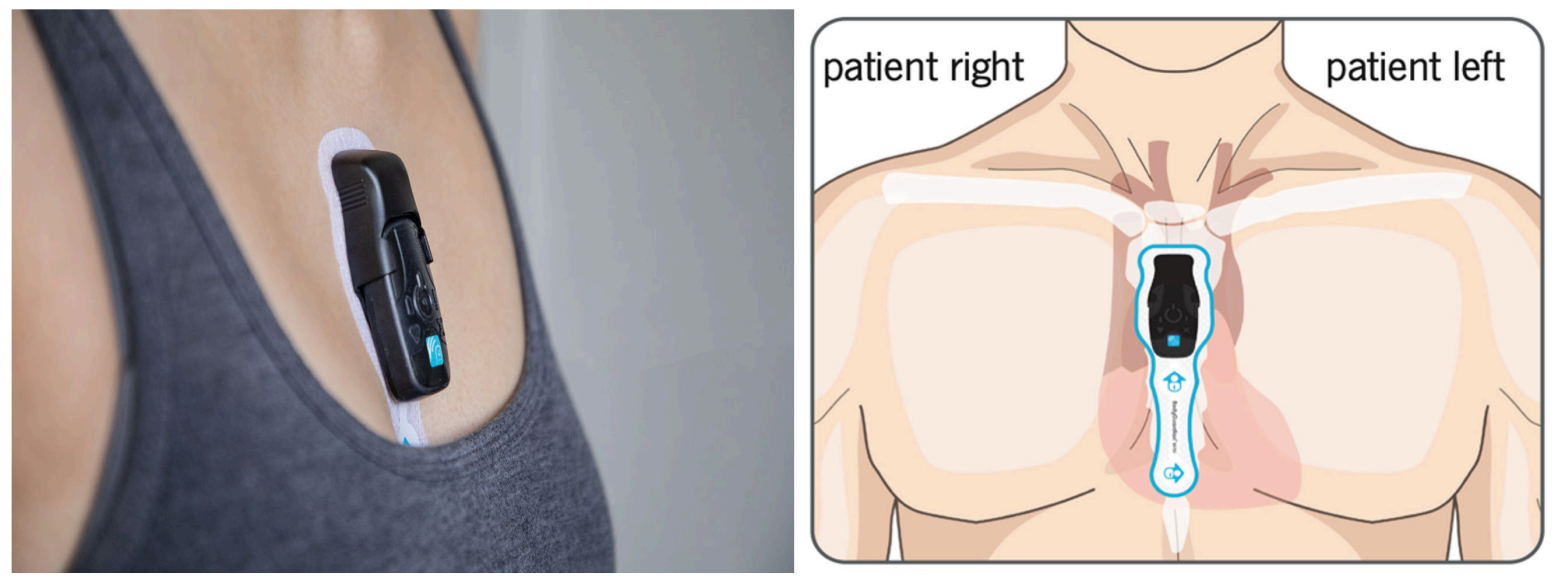
14-day ambulatory heart monitor (Preventice BodyGuardian Mini).

Participants will have an electronic case report form completed at randomisation (table 2). Recruited participants regardless of allocation group, will be referred for syncope assessment as per local service protocol and subsequent investigations will be arranged at the discretion of the treating clinician. Participants randomised to the intervention arm will be fitted with a 14-day ambulatory heart monitor (Preventice BodyGuardian Mini) applied by the study team as soon after randomisation as possible. The ambulatory ECG monitor will be placed on the participant’s chest wall over the sternum, connected directly to an ECG electrode sticker (figure 2). The participant’s skin does not require shaving but is cleaned prior to attaching the device, which are easily removed by the participant after 14 days. The monitor can be worn by both women and men.

**Table 2:** Study assessments

Assessment	Screening	Day 1 baseline	Monthly	90 days	Every 3 months	1 year	2 years
**Window of time for evaluation**	**n/a**	**n/a**	**±14 days**	**±14 days**	**n/a**	**±30 days**	**±30 days**
Assessment of eligibility criteria	X						
Informed consent	X						
eCRF completion including demographic data and contact details	X						
Routine clinical care (eg, ECG)	X						
Randomisation		X					
Intervention group participants fitted with a 14-day ambulatory heart monitor		X					
Referral for syncope assessment and standard care monitoring as per local service protocol to be seen ideally within 4–6 weeks of the index event especially if discharged from ED or if this did not occur during index admission		X					
EQ-5D-5L questionnaires		X				X	X
NHS resource usage data from routine hospital electronic healthcare records extracted by the local study team)				X		X	X
Participant contacted on a 4-weekly basis via automated text message, email or phone with a link to a brief web-based questionnaire asking for the number of syncope events experienced since last response, and the number of GP attendances for any reason			X				
Participants not responding for 3 consecutive months to receive phone call from central study team to collect missing data, ensure no syncope episodes have occurred and to encourage continued future engagement. Participants with a mean of 5 or more episodes/month will also receive a phone call from the central study team to ensure that participants are recording true syncope events and are seeking appropriate medical advice					X		
Participant satisfaction questionnaire						X	

eCRF, electronic case report form; ED, emergency department; GP, general practitioner.

Participants will be required to press the button on the heart monitor after any syncopal event. They will also record symptomatic episodes in a paper diary. The participant will wear the ambulatory ECG monitor for a maximum of 14 days after which they will remove and return it in a prepaid envelope to Preventice UK. The monitor will be reported by a cardiac physiologist, shared with the participant’s local study team, placed in the participant’s health record and shared with the participant’s treating clinician. Treatment of device findings will be at the discretion of the treating clinician at each site. The participant’s general practitioner (GP) will be informed that the participant has been enrolled in the study and will be informed of the results of any ECG investigations via routine hospital clinical correspondence. Participants will be informed of the results of any ECG investigations via routine hospital clinical correspondence. Any study participant with a serious arrhythmia (box 2) on the ECG report will be contacted as soon as possible by the local team and managed appropriately according to local policy.

All participants will be contacted on a 4-weekly basis ±14 days for 2 years via automated text message or email (whichever they prefer) with a link to a brief web-based questionnaire asking for the number of syncope events experienced since their last response and how many of these they attended hospital for. Those who are unable to access digital forms of communication will receive phone calls. They will also be asked since their last response how many times they have visited their GP practice for any reason including all face-to-face, telephone and online consultations. This will import directly into the central ECTU study database. This patient-reported data will be used to inform the primary endpoint. The small number of participants unable to access digital forms of communication and participants not responding for 3 consecutive months will receive a phone call from the central study team (blinded to participant’s study arm allocation) to collect missing data, ensure no syncope episodes have occurred and to encourage continued future engagement. Participants with a mean of 5 or more episodes/month will also receive a phone call from the central study team to ensure that participants are recording true syncope events and are seeking appropriate medical advice.

We were very thoughtful about the potential impact of COVID-19 when designing this study including ensuring ambulatory ECG monitors can be sent directly to patients and designing participant follow-up to ensure no additional research related hospital visits are required.

Participants will also be contacted at one and 2 years±30 days, by the central study team (blinded to participants study arm allocation) to complete a quality-of-life questionnaire. In the event of non-response, participants will be contacted on up to three occasions. The participants’ involvement in the study will cease at 2 years. Endpoint data including NHS resource usage will be extracted by the local study team from routine hospital electronic healthcare records at 90 days, 1 and 2 years and will be entered into a bespoke database. ECTU will collect and clean primary data and perform primary and secondary analyses.

#### Timelines

The study started on 1 August 2021 and the first patient was recruited on 15 July 2022. Recruitment is planned to end on 31 December 2023, the with study completing on 31 July 2025.

#### Sample size

Two thousand two hundred and thirty-four participants (1117 standard and 1117 intervention) presenting acutely with syncope whose syncope remains unexplained after initial ED/AMU assessment will be recruited. Using an estimated mean 1-year recurrence rate in untreated patients of 42.5%^6^ and a reduction in 1-year recurrence rate to 10% in patients who are treated^11 19^ then the hypothesis is a 1-year recurrence rate in the standard group (2% diagnosed with a symptomatic significant arrhythmia with 90% of these expected to not have recurrence after treatment) of 40.7% compared with a recurrence rate of 33.1% in the treatment group (10.5% of pilot participants diagnosed with symptomatic significant arrhythmia at 90 days with 90% of these expected to not have recurrence after cardiac arrhythmia treatment) equating to an event rate ratio of 0.81. A more conservative effect size of 6% (40% vs 34%) will be assumed corresponding to a more conservative event rate ratio of 0.85. A large study of unexplained untreated syncope patients^20^ suggests a median (IQR) number of events in the preceding 2 years of 3 (2–4) with a median (IQR) per year of 2 (1–3.5). The ESC guidelines^6^ suggest that the number of events has a good fit to a Poisson distribution and the untreated event rate postattendance is about 70% reduced from the preattendance rate. The ESC guidelines^6^ and PICTURE^20^ suggest a mean number of events per participant of approximately 1 during 1 year of follow-up. If we assume that this follows a negative binomial distribution (which allows for ‘over dispersion’ vs the Poisson distribution) then a study of 1064 participants per group would have 90% power (two-sided significance level=5%, over dispersion=0.25) to detect an event rate ratio of 0.85.

The study will recruit an extra 5% in each arm (ie, 1117 participants per arm; 2234 in total) to allow for drop-out/loss to follow-up although we expect this to be low (<1% in pilot) and drop out due to death (<1% in pilot). It is expected that most people will respond to some text/email follow-ups, but few will respond to all. We will therefore call any participant who has not responded for 3 consecutive months to ensure no syncope episodes have occurred and to encourage continued future engagement. Participants will be defined as lost to follow-up only if both 1-year electronic patient health record data and 1-year self-reported data are unavailable.

#### Study progression criteria

This study will include an internal recruitment pilot phase with stop-go recruitment milestone criteria to mitigate risk to the funder. This internal pilot will be used to confirm recruitment rates and aims to recruit the first 400 participants (almost one-fifth of the sample size) from 10 sites by the end of study month 13. By the end of study month 13, the aim is to have 400 participants (18%) enrolled with an average recruitment rate/site/month of at least five participants in the best 60% of sites, with at least 10 sites open (table 3).

**Table 3:** Internal recruitment pilot study progression criteria

By end of study month 13	Red	Amber	Green
Total number of participants recruited	≤200	201–399	≥400
Recruitment of total required (%)	9	10–17	18
Average recruitment rate/site/active months in the best 60% of sites^*^	3	4	5
Number of sites open	<5	5–10	>10

*Sites recruitment rate will be calculated from site opening date.

If by the end of study month 19, overall recruitment is less than 1300 participants (58%), OR average recruitment rate/site/month is less than 6 participants in the best 60% of sites, OR there are less than 20 sites recruiting, we will further expand the number of NHS sites recruiting. We will also consider whether study extension is required.

#### Data analysis plan

The primary outcome, number of self-reported episodes of syncope in the 12 months following randomisation, will be analysed by negative binomial regression. The primary outcome event rate ratio (14-day ambulatory heart monitor vs standard care) will be reported with its 95% CI. An offset term for follow-up duration will be included to account for participants with partial follow-up.

The secondary outcomes for the number of syncope episodes at 90 days and 2 years, will be analysed similarly. Binary secondary outcomes will be analysed by logistic regression, reporting the OR (14-day ambulatory heart monitor vs standard care) and its 95% CI. Full details of analysis, including the estimand(s) of interest and methods for handling missing data, will be written into a Statistical Analysis Plan, which will be finalised prior to database lock without knowledge of the unblinded treatment allocations.

#### Cost-effectiveness analysis

Anonymised electronic healthcare record data will be sent to our health economist in Sheffield to apply unit costs and tariffs, to estimate within trial costs and QALYs, and then to undertake lifetime economic modelling. Both within trial and lifetime cost-effectiveness analysis will be performed. In within trial analysis, costs will be estimated by applying national unit costs to items of resource use (monitoring, hospitalisation, treatment, health and social care) to estimate the mean cost per participant in each arm of the trial. Cost-effectiveness will then be estimated as the incremental cost per syncope episode avoided and the incremental cost per QALY gained, with QALYs being estimated from EQ-5D questionnaires. Lifetime cost-effectiveness will be estimated using decision analytic modelling from published sources of life expectancy, annual costs and corresponding annual utilities. This will explore the potential impact of events, such as syncope episode resulting in death or injury, that have consequences beyond the timeframe of the trial.

#### Interim analysis

In addition to the blinded sample size review to ensure that the trial achieves the required statistical power, there will be a single interim futility analysis for the primary outcome performed after the 18th month of recruitment (end of study month 25). At this point 6 months of 1-year follow-up data will be available. We anticipate at least 400 participants will have undergone 12-month follow-up for the primary outcome at this point and will be able to be analysed in this futility analysis.

### Patient and public involvement

The ASPIRED study patient and public involvement group is made up of patient representatives, lay members and a representative from the Arrhythmia Alliance. They have been involved in informing the study research questions and study protocol in particularly the methods and timings of patient follow-up, and the development of all patient facing information.

### Ethics and dissemination

The ASPIRED trial received a favourable ethical opinion from South East Scotland Research Ethics Committee 01 (21/SS/0073).

#### Participant capacity and consent

Capacity will be assessed by the research team or a clinician responsible for the treatment of the participant. The trial excludes patients who have inability to give informed consent and therefore patients with temporary incapacity due to their current illness or with permanent incapacity will not be recruited.

#### Safety considerations

##### Bias

The primary outcome is a quantitative endpoint (number of syncope episodes) collected through automated participant reporting importing directly into the ECTU central study database to reduce reporting bias. Central research staff who phone participants will be blinded to participant allocation.

##### Adverse events

A secondary endpoint for the study is serious outcomes at 90 days, 1 and 2 years. This data will therefore be routinely collected as part of the study and not recorded as an adverse event (AE). Hospital admission will also not be recorded as an AE. The only AEs recorded will be those directly related to the use of initial ambulatory ECG recording both in the intervention and standard care groups. Participants will be asked through the automated monthly email/text questionnaire at month 2, whether they suffered any complications related to wearing any monitoring devices in the first 2 months of the trial.

A Trial Steering Committee (TSC) will be established to oversee the conduct and progress of the trial. The details of the TSC will be captured in a separate charter. An independent Data Monitoring Committee (DMC) will be established to oversee the safety of participants in the trial. The details of the DMC will be captured in a separate charter.

##### Data management

Identifiable data collected by the study will not be transferred to any external individuals or organisations outside of the sponsoring organisations.

##### Study dissemination

We will disseminate the results of this study widely through high impact peer-reviewed publications, presentations at international conferences, local and national websites, charity newsletters and websites and media outlets such as television and radio. We will also share our results through specific interest groups such as Arrhythmia Alliance and disseminate findings among guideline development groups such as ESC, SIGN, NICE and American College of Cardiology.

## Supplementary Material

Reviewer comments

Author's manuscript
