# Supplementary material for: Multicentre open label randomised controlled trial of immediate enhanced ambulatory ECG monitoring versus standard monitoring in acute unexplained syncope patients: the ASPIRED study
Source: BMJ Open. 2023 Feb 23;13(2):e069530. doi: 10.1136/bmjopen-2022-069530 (PMC9950891; doi:10.1136/bmjopen-2022-069530)
Supplement: Author's manuscript [file bmjopen-2022-069530.draft_revisions.pdf]

BMJ Open is committed to open peer review. As part of this commitment we make the peer review history of every article we publish publicly available.

When an article is published we post the peer reviewers' comments and the authors' responses online. We also post the versions of the paper that were used during peer review. These are the versions that the peer review comments apply to.

The versions of the paper that follow are the versions that were submitted during the peer review process. They are not the versions of record or the final published versions. They should not be cited or distributed as the published version of this manuscript.

BMJ Open is an open access journal and the full, final, typeset and author-corrected version of record of the manuscript is available on our site with no access controls, subscription charges or pay-per-view fees (http://bmjopen.bmj.com).

If you have any questions on BMJ Open's open peer review process please email info.bmjopen@bmj.com

BMJ Open

# Multi-centre open label randomised controlled trial of immediate enhanced ambulatory ECG monitoring versus standard monitoring in acute unexplained syncope patients: The ASPIRED study.



| | |
|---:|---|
| Journal: | *BMJ Open* |
| Manuscript ID | bmjopen-2022-069530 |
| Article Type: | Protocol |
| Date Submitted by the Author: | 25-Oct-2022 |





| | |
|---:|---|
| Complete List of Authors: | Reed, Matthew; The University of Edinburgh Usher Institute of Population Health Sciences and Informatics, Emergency Medicine Research Group Edinburgh (EMERGE)<br>Grubb, Neil; Edinburgh Royal Infirmary, Department of Cardiology<br>Lang, Chris; Edinburgh Royal Infirmary, Department of Cardiology<br>Goodacre, Steve; The University of Sheffield, Medical Care Research Unit<br>O'Brien, Rachel; Edinburgh Royal Infirmary, Emergency Medicine Research Group Edinburgh (EMERGE)<br>Weir, Christopher; The University of Edinburgh Usher Institute of Population Health Sciences and Informatics, Edinburgh Clinical Trials Unit, Usher Institute<br>Thokala, Praveen; The University of Sheffield<br>Freeman, Nicola; Edinburgh Royal Infirmary, Emergency Medicine Research Group Edinburgh (EMERGE)<br>Blackstock, Caroline; Edinburgh Royal Infirmary, Emergency Medicine Research Group Edinburgh (EMERGE)<br>Dinsmore, Lynn; The University of Edinburgh Usher Institute of Population Health Sciences and Informatics, Edinburgh Clinical Trials Unit, Usher Institute<br>Boyd, Julia; The University of Edinburgh Usher Institute of Population Health Sciences and Informatics, Edinburgh Clinical Trials Unit<br>Adamestam, Imad; The University of Edinburgh Usher Institute of Population Health Sciences and Informatics, Edinburgh Clinical Trials Unit, Usher Institute<br>Macrae, Pam; ASPIRED study Patient Advisory Group<br>Hannigan, Robert; ASPIRED study Patient Advisory Group<br>Lobban, Trudie; Arrhythmia Alliance |





| | |
|---:|---|
| Keywords: | ACCIDENT & EMERGENCY MEDICINE, Adult cardiology < CARDIOLOGY, Pacing & electrophysiology < CARDIOLOGY, GENERAL MEDICINE (see Internal Medicine), Cardiology < INTERNAL MEDICINE |
| | |

SCHOLARONE™
Manuscripts

**Title:** Multi-centre open label randomised controlled trial of immediate enhanced ambulatory ECG monitoring versus standard monitoring in acute unexplained syncope patients: The ASPIRED study.

**Authors:** Matthew J Reed [1,2], Neil Grubb [3], Chris Lang [3], Steve Goodacre [4], Rachel O'Brien [1], Christopher Weir [5], Praveen Thokala [4], Nicola Freeman [1], Caroline Blackstock [1], Lynn Dinsmore [5], Julia Boyd [5], Imad Adamestam [5], Pam Macrae [6], Robert Hannigan [6], Trudie Lobban [6]

1. Emergency Medicine Research Group Edinburgh (EMERGE), Royal Infirmary of Edinburgh, Edinburgh, UK
2. Acute Care Edinburgh, Usher Institute, University of Edinburgh, Edinburgh, UK
3. Department of Cardiology, Royal Infirmary of Edinburgh, Edinburgh, UK
4. School of Health and Related Research (ScHARR), University of Sheffield
5. Edinburgh Clinical Trials Unit (ECTU), University of Edinburgh, Edinburgh, UK
6. ASPIRED study Patient Advisory Group

**Corresponding Author**
Professor Matthew J Reed, Emergency Medicine Research Group Edinburgh (EMERGE), Department of Emergency Medicine, Royal Infirmary of Edinburgh, Edinburgh, UK.

Email: mattreed@ed.ac.uk
Tel: +44 (0) 131 242 1284

**Trial Sponsor:** The University of Edinburgh and Lothian Health Board, ACCORD, The Queen's Medical Research Institute, 47 Little France Crescent, Edinburgh, EH16 4TJ, UK.

**Word Count:** 4417 (4,000) words, 20 references, 2 Figures, 5 Tables

**Abstract Word Count:** 198

For peer review only

**ABSTRACT:**

**Introduction:** Diagnosing underlying arrhythmia in Emergency Department (ED) syncope patients is difficult. There is evidence that diagnostic yield for detecting underlying arrhythmia is highest when cardiac monitoring devices are applied early, ideally at the index visit **[1-3].** This strategy has the potential to change current syncope management from low diagnostic yield Holter to higher yield ambulatory monitoring, reduce episodes of syncope, reduce risk of recurrence and its potential serious consequences, reduce hospital admissions, reduce overall health costs and increase quality of life by allowing earlier diagnosis, treatment and exclusion of clinically important arrhythmias.

**Methods and analyses:** This is a UK open prospective parallel group multicentre randomised controlled trial of an immediate 14-day ambulatory patch heart monitor versus standard care in 2234 patients presenting acutely with unexplained syncope. Our patient focussed primary endpoint will be number of episodes of syncope at one year. Health economic evaluation will estimate the incremental cost per syncope episode avoided and quality-adjusted life year gained.

**Ethics and dissemination:** Informed consent for participation will be sought. The ASPIRED trial received a favourable ethical opinion from South East Scotland Research Ethics Committee 01 (21/SS/0073). Results will be disseminated via scientific publication, lay summary, and visual abstract.



**Registration Details:** ISRCTN 10278811 - https://doi.org/10.1186/ISRCTN10278811
Protocol version 3; 14 March 2022


**ARTICLE SUMMARY**

**Strengths & Limitations of this study**

- The ASPIRED trial uses non-blinded randomisation to compare immediate enhanced ambulatory ECG monitoring versus standard monitoring in acute unexplained syncope patients.
- The primary outcome measure is a patient focussed measure very relevant to the population of patients who suffer syncope.
- We are using health economic analysis and a separate embedded qualitative study to assess the wider implications of using immediate enhanced ambulatory ECG monitoring in this population.
- In contrast to the few published studies on ambulatory monitoring in syncope patients which are generally single centred, without any comparative group and with heterogeneous patient populations and device capabilities, this study is multicentred and includes a standard care group.


**Key Words:** Ambulatory Electrocardiography Monitoring, Cardiac Arrhythmias, and Syncope, Diagnostic Tests


For peer review only

# INTRODUCTION

## Background and Rationale

Syncope (or blackout) is common; 650,000 patients present to UK EDs every year **[4]**. The 3 underlying causes are neurocardiogenic (including simple faint), postural hypotension (blood pressure fall on standing) and cardiac disease (structural heart disease or cardiac arrhythmia). Diagnosis is difficult and is not made in around 50% of patients after assessment **[4]**. Whilst vasovagal and postural syncope are relatively benign, serious pathology (affecting 7 out of every 100 patients at one month after ED attendance **[5]**) include arrhythmia (an abnormal heart rhythm). When cardiac arrhythmias are detected, they are most commonly asystolic pauses, reflex bradycardia or advanced atrioventricular block, with tachycardia being the minority **[6].**

The difficulty in the ED is to differentiate between the causes of syncope and identify patients at higher risk. This can be complicated as many patients have fully recovered on ED arrival and their examination and presenting electrocardiogram (ECG) may both be normal. The current method for establishing cardiac arrhythmia as the cause of syncope rests on correlating the arrhythmia with symptoms. The lack of efficacy and availability of commonly used monitoring devices means most high and medium risk patients are admitted to hospital for observation and telemetry (if available), with escalating costs **[1]**. Unfortunately, many (around 50% of patients after assessment) still end up being discharged without a diagnosis **[7]**.

In general, syncope reoccurs in around 50% of patients within a year. Recurring episodes increase hospital admissions, health costs and importantly reduce the quality of life of patients. Whilst there is a wide variation in the literature with respect to the number of syncope episodes and recurrence rates pre and post treatment **[6, 8-10]** once a cardiac arrhythmia diagnosis is made and treatment initiated, around only 10% of patients will have a 1-year recurrence **[11]** and syncope episodes will drop by over 90% **[11]**.

There is evidence that the diagnostic yield for detecting underlying arrhythmia is highest when cardiac monitoring devices are applied early after syncope, ideally at the index visit **[1-3]**. To solve the problems with current routine ECG monitoring devices, several novel ambulatory devices have recently been developed **[1, 12-15]**. These devices are non-invasive, water-resistant, have no leads or wires, are discreet to wear and are CE-marked for clinical use in the UK. They continuously monitor the heart for up to 14 days including during sleep, in the shower, and during moderate exercise and some have a button for patients to capture symptomatic events. They offer medium duration high fidelity ECG recording and are well tolerated **[16,17]**. At the end of the monitoring period, patients return the devices back to the company (by mail) or NHS (to be downloaded) for analysis. The PATCH-ED pilot study helped establish trial methods and numbers of available participants informing this RCT **[1]**. This pilot study showed a novel ECG monitoring device was able to detect serious cardiac arrhythmia

For peer review only

requiring treatment in a significant proportion of patients and demonstrated the potential to influence clinical management decisions relating to hospital admission and participant outcomes.

The ASPIRED randomised controlled trial (RCT) will compare a novel ambulatory cardiac monitoring device with standard practice in syncope patients. We hypothesise that applying cardiac monitoring early after syncope at the index visit is the optimum strategy to detect, diagnose, treat, and exclude underlying cardiac arrhythmia.

**Study Objectives:**

**Primary:** To determine whether immediate, enhanced (14-day) ambulatory ECG monitoring decreases the number of self-reported episodes of syncope at one year compared to standard care monitoring in acute unexplained syncope patients.

**Secondary:** To determine whether immediate, enhanced (14-day) ambulatory ECG monitoring in acute unexplained syncope patients can:

1. Decrease the number of episodes of self-reported syncope at 90 days, and 2 years compared to standard care monitoring.
2. Decrease the time to detection of clinically significant cardiac arrhythmia compared to standard care monitoring.
3. Increase the rate of detection of clinically significant cardiac arrhythmia at 90 days and 1 year compared to standard care monitoring.
4. Increase the rate of ECG/symptom correlation at 90 days and 1 year compared to standard care monitoring.
5. Demonstrate cost effectiveness compared to standard care monitoring.
6. Decrease the number of episodes of syncope identified in the medical records at 90 days, 1 and 2 years compared to standard care monitoring.
7. Decrease the index hospital admission rate and duration of hospital stay compared to standard care monitoring
8. Decrease 90 days, 1- and 2-year syncope recurrence rates (identified in the medical records and self-reported) compared to standard care monitoring
9. Increase patient satisfaction compared to standard care monitoring
10. Decrease the rate of 30 day, 1 and 2 year all cause death compared to standard care monitoring
11. In the intervention group, by reporting the timing of detection of clinically significant cardiac arrhythmia what is the optimum duration of acute ambulatory ECG monitoring
12. Increase the affect rate of diagnostic testing and therapeutic intervention.

For peer review only

## METHODS AND ANALYSIS

### Study design

This is a UK open prospective parallel group multicentre RCT of an immediate 14-day ambulatory patch heart monitor versus standard care monitoring in 2234 participants presenting acutely with unexplained syncope. The patient focussed primary endpoint will be number of episodes of syncope at one year **[Table 1]**.

**Study Eligibility:** 2234 adult (16 years or older) participants presenting acutely to UK hospitals with syncope remaining unexplained after initial ED or Acute Medicine Unit (AMU) assessment will be enrolled. Syncope will be defined as transient loss of consciousness (TLOC) with inability to maintain postural tone and immediate complete spontaneous recovery without medical intervention **[18]**.

**Inclusion Criteria:**

1. Syncope remains unexplained after initial ED/AMU assessment.
2. Aged ≥ 16 years
3. Patient has capacity
4. Local resident (i.e. resident within local health board so will not be lost to medical record follow up)
5. <5 self-reported episodes of syncope in the previous month

**Exclusion Criteria:**

1. Obvious underlying cause after assessment
    a. Features of vasovagal syncope **(see Table 2) AND** absence of structural heart disease **AND** normal physical examination **AND** normal ECG
    b. Arrhythmia on pre-hospital or hospital ECG as likely cause of syncope
    c. Postural hypotension (symptomatic postural drop >20 mmHg **AND** suggestive history)
    d. Confirmed diagnosis of Pulmonary Embolus or Acute Myocardial Infarction
    e. Radiological diagnosis or clinical signs/symptoms of cerebrovascular accident/transient ischemic attack or subarachnoid haemorrhage
    f. Evidence of:
        i. Haemorrhage
        ii. Alcohol or illicit drugs
        iii. Epileptic seizure
        iv. Hypoglycemia
        v. Head trauma
        vi. Other obvious cause of syncope as presumptive cause of TLoC
2. Inability to consent
3. Previous recruitment into the study
4. Patient in custody or prison
5. Aged <16 years

For peer review only

6. Patient does not reside within local health board and will therefore be lost to medical record follow up
7. 5 or more self-reported episodes of syncope in previous 4 weeks

Pregnancy is not an exclusion criteria.

**Participant Recruitment:** Recruitment will take place in around 30-40 NHS acute tertiary and district hospitals. **Figure 1** summarises the study design and participant recruitment**.**

**Randomisation and Interventions**

Randomisation will be performed using a web-based randomisation service to ensure allocation concealment, managed by Edinburgh Clinical Trials Unit (ECTU). The allocation sequence will be created by an ECTU database programmer using computer-generated pseudo-random numbers. Participants will be randomised, 1:1, between the two study arms. Stratification by site will be used to ensure there will not be a significant imbalance between the number randomised to intervention and control at any site. Stratification by other site-level characteristics will not be performed.

Standard care will include all care usually given to unexplained syncope patients at each participating site along with some form of standard care monitoring such as but not limited to wired inpatient telemetry, Holter style monitoring or implantable ECG recorder. The study will be conducted over 4 years. Recruitment will take place over 18 months. Intervention group participants will be fitted with a 14-day ambulatory heart monitor. All participants will be followed-up for 2 years after index event.

**Outcome Measures:**

**Primary Endpoint:** Number of self-reported episodes of syncope at one year.

**Secondary Endpoints:**

1. Within trial cost effectiveness (cost per syncope avoided and cost per quality adjusted life year [QALY] gained), and lifetime cost per QALY gained at (a) 1 year and (b) 2 years
2. Number of self-reported episodes of syncope at (a) 90 days and (b) 2 years, those identified in the medical records at (c) 90 days, (d) 1 year and (e) 2 years, and syncope recurrence rate at (f) 90 days, (g) 1 year and (h) 2 years
3. Index presentation hospital (a) admission rate and (b) duration of hospital stay
4. Patient Satisfaction (measured using a patient questionnaire) at 1 year
5. Clinically significant cardiac arrhythmia (Serious and/or symptomatic cardiac arrhythmia **Table 3**) at (a) 90-days, (b) 1 year and (c) 2 years.
6. (a) 30 day, (b) 1 year and (c) 2 year all cause death
7. Detection of diagnostic ECG/symptom correlation (symptomatic) at (a) 90-days, (b) 1 year and (c) 2 years.
8. Time to detect clinically significant cardiac arrhythmia (i.e. time to clinician being aware)

9. In the intervention group, duration of enhanced ambulatory ECG monitoring required to detect clinically significant cardiac arrhythmia
10. Number and type of diagnostic tests and therapeutic interventions at (a) 1 year and (b) 2 years

**Trial assessments**

**Participant identification:** Potential participants will be identified from EDs/AMUs or other acute settings and approached either during their ED/AMU or hospital stay or contacted after discharge and invited to take part in the study.

**Participant consent:** Potential participants will be given a Participant Information Sheet (PIS). Potential participants will be randomised within 72 hours of their hospital attendance. If a patient has been discharged from hospital, then they will be contacted by the local clinical team or research team (if part of the clinical team), and a copy of the PIS will be emailed or posted out to the patient. The PIS will also be available on the trial website. A delegated member of the local study team will seek verbal consent over the telephone and will sign the consent form on behalf of the participant. The original consent form will be filed in the Investigator Site File (ISF) and a copy will be sent to the participant. Alternatively, the patient can attend the hospital with travel expenses, to provide written informed consent in person. The ambulatory ECG device can be sent to the participant's address or collected from the local study team if randomised to the intervention arm. Each participating centre will upload screening information of non-identifiable potentially eligible patients who were approached to participate in the study, onto the study database. Participants are free to withdraw from the study at any point or can be withdrawn by the Investigator

Participants will have an eCRF completed at randomisation **[Table 4]**. Recruited participants regardless of allocation group, will be referred for syncope assessment as per local service protocol and subsequent investigations will be arranged at the discretion of the treating clinician. Participants randomised to the intervention arm will be fitted with a 14-day ambulatory heart monitor (Preventice BodyGuardian Mini) applied by the study team as soon after randomisation as possible. The ambulatory ECG monitor will be placed on the participant's chest wall over the sternum, connected directly to an ECG electrode sticker **[Figure 2]**. The participant's skin does not require shaving but is cleaned prior to attaching the device, which are easily removed by the participant after 14 days. The monitor can be worn by both women and men.

Participants will be required to press the button on the heart monitor after any syncopal event. They will also record symptomatic episodes in a paper diary. The participant will wear the ambulatory ECG monitor for a maximum of 14 days after which they will remove and return it in a pre-paid envelope to Preventice UK. The monitor will be reported by a Cardiac Physiologist, shared with the participant's local study team, placed in the participant's health

For peer review only

record, and shared with the participant's treating clinician. Treatment of device findings will be at the discretion of the treating clinician at each site. The participant's GP will be informed that the participant has been enrolled in the study and will be informed of the results of any ECG investigations via routine hospital clinical correspondence. Participants will be informed of the results of any ECG investigations via routine hospital clinical correspondence. Any study participant with a serious arrhythmia **[Table 3]** on the ECG report will be contacted as soon as possible by the local team and managed appropriately according to local policy.

All participants will be contacted on a 4-weekly basis +/- 14 days for 2 years via automated text message or email (whichever they prefer) with a link to a brief web-based questionnaire asking for the number of syncope events experienced since their last response and how many of these they attended hospital for. Those who are unable to access digital forms of communication will receive phone calls. They will also be asked since their last response how many times they have visited their GP practice for any reason including all face-to-face, telephone and online consultations. This will import directly into the central ECTU study database. This patient reported data will be used to inform the primary endpoint. The small number of participants unable to access digital forms of communication and participants not responding for 3 consecutive months will receive a phone call from the central study team (blinded to participant's study arm allocation) to collect missing data, ensure no syncope episodes have occurred and to encourage continued future engagement. Participants with a mean of 5 or more episodes/month will also receive a phone call from the central study team to ensure that participants are recording true syncope events and are seeking appropriate medical advice.

Participants will also be contacted at one and two years +/- 30 days, by the central study team (blinded to participants study arm allocation) to complete a quality-of-life questionnaire. In the event of non-response, participants will be contacted on up to 3 occasions. The participants' involvement in the study will cease at 2 years. Endpoint data including NHS resource utilisation will be extracted by the local study team from routine hospital electronic healthcare records at 90 days, 1 and 2 years and will be entered into a bespoke database. ECTU will collect and clean primary data and perform primary and secondary analyses.

**Sample Size**

2234 participants (1117 standard and 1117 intervention) presenting acutely with syncope whose syncope remains unexplained after initial ED/AMU assessment will be recruited. Using an estimated mean 1-year recurrence rate in untreated patients of 42.5% **[6; Sup Table 10]** and a reduction in 1-year recurrence rate to 10% in patients who are treated **[11,19]** then the hypothesis is a 1-year recurrence rate in the standard group (2% diagnosed with a symptomatic significant arrhythmia with 90% of these expected to not have recurrence after treatment) of 40.7% compared to a recurrence rate of 33.1% in the treatment group (10.5% of pilot participants diagnosed with symptomatic significant arrhythmia at 90 days with 90%

For peer review only

of these expected to not have recurrence after cardiac arrhythmia treatment) equating to an event rate ratio of 0.81. A more conservative effect size of 6% (40% vs 34%) will be assumed corresponding to a more conservative event rate ratio of 0.85. A large study of unexplained untreated syncope patients **[20]** suggests a median (IQR) number of events in the preceding 2 years of 3 (2-4) with a median (IQR) per year of 2 (1-3.5). The ESC Guidelines **[6]** suggest that the number of events has a good fit to a Poisson distribution and the untreated event rate post-attendance is about 70% reduced from the pre-attendance rate. The ESC guidelines **[6]** and PICTURE **[20]** suggest a mean number of events per participant of approximately 1 during 1 year of follow-up. If we assume that this follows a negative binomial distribution (which allows for "over dispersion" versus the Poisson distribution) then a study of 1064 participants per group would have 90% power (two-sided significance level=5%, over dispersion=0.25) to detect an event rate ratio of 0.85.

The study will recruit an extra 5% in each arm (i.e. 1117 participants per arm; 2234 in total) to allow for drop-out/loss to follow up although we expect this to be low (<1% in pilot) and drop out due to death (<1% in pilot). It is expected that most people will respond to some text/email follow-ups, but few will respond to all. We will therefore call any participant who has not responded for 3 consecutive months to ensure no syncope episodes have occurred and to encourage continued future engagement. Participants will be defined as lost to follow up only if both one-year electronic patient health record data and one-year self-reported data is unavailable.

**Study Progression Criteria:** This study will include an internal recruitment pilot phase with stop-go recruitment milestone criteria to mitigate risk to the funder. This internal pilot will be used to confirm recruitment rates and aims to recruit the first 400 participants (almost one fifth of the sample size) from 10 sites by the end of study month 13. By the end of study month 13, the aim is to have 400 participants (18%) enrolled with an average recruitment rate/site/month of at least 5 participants in the best 60% of sites, with at least 10 sites open **[Table 5].**

If by the end of study month 19, overall recruitment is less than 1300 participants (58%), OR average recruitment rate/site/month is less than 6 participants in the best 60% of sites, OR there are less than 20 sites recruiting, we will further expand the number of NHS sites recruiting. We will also consider whether study extension is required.

**Data Analysis Plan:** The primary outcome, number of self-reported episodes of syncope in the 12 months following randomisation, will be analysed by negative binomial regression. The primary outcome event rate ratio (14-day ambulatory heart monitor vs standard care) will be reported with its 95% confidence interval. An offset term for follow-up duration will be included to account for participants with partial follow up.

For peer review only

The secondary outcomes for the number of syncope episodes at 90 days and 2 years, will be analysed similarly. Binary secondary outcomes will be analysed by logistic regression, reporting the odds ratio (14-day ambulatory heart monitor vs standard care) and its 95% confidence interval. Full details of analysis, including the estimand(s) of interest and methods for handling missing data, will be written into a Statistical Analysis Plan, which will be finalised prior to database lock without knowledge of the unblinded treatment allocations.

**Cost Effectiveness Analysis:** Anonymised electronic healthcare record data will be sent to our health economist in Sheffield to apply unit costs and tariffs, to estimate within trial costs and QALYs, and then to undertake lifetime economic modelling. Both within trial and lifetime cost-effectiveness analysis will be performed. In within trial analysis, costs will be estimated by applying national unit costs to items of resource use (monitoring, hospitalisation, treatment, health, and social care) to estimate the mean cost per participant in each arm of the trial. Cost-effectiveness will then be estimated as the incremental cost per syncope episode avoided and the incremental cost per quality-adjusted life year (QALY) gained, with QALYs being estimated from EQ5D questionnaires. Lifetime cost-effectiveness will be estimated using decision analytic modelling from published sources of life expectancy, annual costs and corresponding annual utilities. This will explore the potential impact of events, such as syncope episode resulting in death or injury, that have consequences beyond the timeframe of the trial.

**Interim analysis:** In addition to the blinded sample size review to ensure that the trial achieves the required statistical power, there will be a single interim futility analysis for the primary outcome performed after the 18th month of recruitment (end of study month 25). At this point 6 months of 1 year follow-up data will be available. We anticipate at least 400 participants will have undergone 12 months follow-up for the primary outcome at this point and will be able to be analysed in this futility analysis.

**Patient and Public Involvement (PPI):** The ASPIRED study PPI group is made up of patient representatives, lay members and a representative from the Arrhythmia Alliance. They have been involved in informing the study research questions and study protocol in particularly the methods and timings of patient follow-up, and the development of all patient facing information.

For peer review only

## ETHICS AND DISSEMINATION

The ASPIRED trial received a favourable ethical opinion from South East Scotland Research Ethics Committee 01 (21/SS/0073).

**Participant Capacity and Consent:** Capacity will be assessed by the research team or a clinician responsible for the treatment of the participant. The trial excludes patients who have inability to give informed consent and therefore patients with temporary incapacity due to their current illness or with permanent incapacity will not be recruited.

**Safety Considerations:**

**Bias:** The primary outcome is a quantitative endpoint (number of syncope episodes) collected through automated participant reporting importing directly into the ECTU central study database to reduce reporting bias. Central research staff who phone participants will be blinded to participant allocation.

**Adverse events:** A secondary endpoint for the study is serious outcomes at 90-days, 1 and 2 years. This data will therefore be routinely collected as part of the study and not recorded as an Adverse Event (AE). Hospital admission will also not be recorded as an AE. The only AEs recorded will be those directly related to the use of initial ambulatory ECG recording both in the intervention and standard care groups. Participants will be asked through the automated monthly email/text questionnaire at month 2, whether they suffered any complications related to wearing any monitoring devices in the first 2 months of the trial.

A Trial Steering Committee (TSC) will be established to oversee the conduct and progress of the trial. The details of the TSC will be captured in a separate charter. An independent Data Monitoring Committee (DMC) will be established to oversee the safety of participants in the trial. The details of the DMC will be captured in a separate charter.

**Data Management:** Data collected or generated by the study (including personal data) will not be transferred to any external individuals or organisations outside of the Sponsoring organisations including Preventice Solutions.

**Study Dissemination:** We will disseminate the results of this study widely through high impact peer-reviewed publications, presentations at international conferences, local and national websites, charity newsletters and websites and media outlets such as television and radio. We will also share our results through specific interest groups such as Arrhythmia alliance and disseminate findings amongst guideline development groups such as ESC, SIGN, NICE and American College of Cardiology (ACC).

**Registration Details**:

Registered at ISRCTN (10278811 https://doi.org/10.1186/ISRCTN10278811)

## AUTHOR CONTRIBUTIONS

For peer review only

All authors contributed to the conception and design of the work, drafting the work, and revising it critically for important intellectual content, final approval of the version to be published, and agree to be accountable for all aspects of the work in ensuring that questions related to the accuracy or integrity of any part of the work are appropriately investigated and resolved.

**ACKNOWLEDGEMENTS**

We thank all patients, lay members, and members of patient and public involvement groups who gave feedback to improve the study design.

**FUNDING STATEMENT**

This work was supported by the British Heart Foundation (BHF) grant number CS/F/20/190016. The funder and sponsor have reviewed the study design and protocol but have no role in the collection, management, analysis, and interpretation of data; writing of the report or the decision to submit the paper for publication.

**COMPETING INTERESTS STATEMENT**

MJR is supported by an NHS Research Scotland Career Researcher Clinician award. All other authors declared no competing interests.

**DATA STATEMENT**

All study data may be available later, on request.

**FIGURE and TABLE LEGENDS**

**Figure 1:** Schematic diagram of the study design and Participant Recruitment

**Figure 2:** 14-day ambulatory heart monitor (Preventice BodyGuardian Mini)

**Table 1:** Study summary in PICO format

**Table 2:** Features of vasovagal / postural syncope

**Table 3:** Definitions of clinically significant cardiac arrhythmias

**Table 4:** Study Assessments

**Table 5:** Internal recruitment pilot study progression criteria

For peer review only

**Table 1:** Study summary in PICO format

| | |
|---|---|
| **P: Population** | Adults presenting acutely to UK hospitals with syncope remaining unexplained after initial ED/AMU assessment |
| **I: Intervention** | 14-day ambulatory heart monitor placed on patients |
| **C: Comparator** | Standard care monitoring |
| **O: Primary Outcome** | Number of self-reported episodes of syncope at one year |

**Table 2:** Features of vasovagal / postural syncope

| |
|---|
| Associated with typical symptoms of reflex syncope (e.g. light-headedness, feeling of warmth, nausea, vomiting) |
| After sudden unexpected unpleasant sight, sound, smell, or pain |
| In association with micturition, defaecation, cough, laughter, venepuncture, blood phobia |
| After prolonged standing or crowded, hot places |
| During a meal or after eating a meal |
| With head rotation or pressure on carotid sinus (e.g. tumour, shaving, tight collars) |
| Associated with standing up quickly from a sitting or lying position |
| Long history (years) of recurrent syncope with low-risk features with the same characteristics of the current episode |

For peer review only

**Table 3:** Definitions of clinically significant cardiac arrhythmias

| |
|---|
| Ventricular Fibrillation (VF) * |
| Ventricular Tachycardia (VT) ≥120 beats per minute (bpm) for ≥30 seconds * |
| VT ≥120 beats per minute for <30 seconds (≥4 beats) * |
| Complete or 3rd degree heart block * |
| Second degree atrioventricular heart block Mobitz type II * |
| Second degree atrioventricular heart block Mobitz type I |
| Pause ≥6 seconds * |
| Sinus pause ≥2.5 seconds when awake or ≥4 seconds at night (but <6 seconds) |
| Sinus bradycardia <30 beats/minute * |
| Bradycardia <40 beats per minute for ≥30 seconds * |
| Bradycardia <40 beats per minute for <30 seconds |
| Sick sinus syndrome with alternating sinus bradycardia and tachycardia |
| Junctional / idioventricular rhythm ≥30 seconds in duration |
| Supraventricular tachycardia >100 beats per minute ≥30 seconds in duration |
| Atrial flutter/fibrillation with ventricular rate >100 bpm or <60 bpm ≥30 seconds in |
| New Atrial flutter/fibrillation ≥30 seconds in duration |

* Defined as 'Serious' clinically significant cardiac arrhythmia

Nb. All arrhythmias will also be classed as symptomatic or asymptomatic during monitoring period

For peer review only

**Table 4: Study Assessments**

| Assessment | Screening | Day 1 baseline | Monthly | 90 days | Every 3 months | 1 year | 2 years |
|---|---|---|---|---|---|---|---|
| **Window of time for evaluation** | n/a | n/a | +/- 14 days | +/- 14 days | n/a | +/- 30 days | +/- 30 days |
| Assessment of Eligibility Criteria | X | | | | | | |
| Informed consent | X | | | | | | |
| eCRF completion including demographic data and contact details | X | | | | | | |
| Routine clinical care (e.g. ECG) | X | | | | | | |
| Randomisation | | X | | | | | |
| Intervention group participants fitted with a 14-day ambulatory heart monitor | | X | | | | | |
| Referral for syncope assessment and standard care monitoring as per local service protocol to be seen ideally within 4-6 weeks of the index event especially if discharged from ED or if this did not occur during index admission. | | X | | | | | |
| EQ-5D-5L questionnaires | | X | | | | X | X |
| NHS resource utilisation data from routine hospital electronic healthcare records extracted by the local study team) | | | | X | | X | X |
| Participant contacted on a 4-weekly basis via automated text message, email or phone with a link to a brief web-based questionnaire asking for the number of syncope events experienced since last response, and the number of GP attendances for any reason. | | | X | | | | |
| Participants not responding for 3 consecutive months to receive phone call from central study team to collect missing data, ensure no syncope episodes have occurred and to encourage continued future engagement. Participants with a mean of 5 or more episodes/month will also receive a phone call from the central study team to ensure that participants are recording true syncope events and are seeking appropriate medical advice. | | | | | X | | |
| Participant satisfaction questionnaire | | | | | | X | |

For peer review only

**Table 5:** Internal recruitment pilot study progression criteria

| **By end of study month 13** | **Red** | **Amber** | **Green** |
|---|---|---|---|
| Total number of participants recruited | ≤200 | 201-399 | ≥400 |
| % recruitment of total required | 9% | 10-17% | 18% |
| Average recruitment rate/site/active months in the best 60% of sites * | 3 | 4 | 5 |
| Number of sites open | <5 | 5-10 | >10 |

* Sites recruitment rate will be calculated from site opening date.

For peer review only

**REFERENCES**

**[1]** Reed MJ, Grubb NJ, Lang CC, Gray AJ, Simpson K, MaCraild AJ, Weir CJ. Diagnostic yield of an ambulatory patch monitor in patients with unexplained syncope after initial evaluation in the Emergency Department: The PATCH-ED study. *Emerg Med J* 2018; 35:477–485.

**[2]** Locati ET, Moya A, Oliveira M, et al. External prolonged electrocardiogram monitoring in unexplained syncope and palpitations: results of the SYNARR-Flash study. Europace 2016; 18: 1265–1272

**[3]** Thiruganasambandamoorthy V, Rowe BH, Sivilotti MLA, et al. Duration of Electrocardiographic Monitoring of Emergency Department Patients With Syncope. Circulation 2019; 139: 1396–1406

**[4]** Reed MJ, Newby DE, Coull AJ et al. Risk stratification Of Syncope in the Emergency department: The ROSE study. *J American College Cardiology* 2010;55(8): 713-721.

**[5]** Reed MJ, Henderson SS, Newby DE, et al. One-year prognosis after syncope and the failure of the ROSE decision instrument to predict 1-year adverse events. *Ann Emerg Med* 2011; 58(3): 250-6.

**[6]** Brignole M, Reed MJ et al. 2018 ESC Guidelines for the diagnosis and management of syncope. *European Heart Journal* 2018; 39(21): 1883-1948; doi:10.1093/eurheartj/ehy037

**[7]** Tattersall L, Reed MJ. The Inpatient Management of Syncope. Emerg Med J. 2010; 27: 870-872.

**[8]** Solbiati M, Costantino G, Casazza G et al. Implantable loop recorder versus conventional diagnostic workup for unexplained recurrent syncope. Cochrane Database Syst Rev.2016; doi: 10.1002/14651858.CD011637.pub2.

**[9]** Farwell DJ, Freemantle N, Sulke N. The clinical impact of implantable loop recorders in patients with syncope. European Heart Journal 2006; 27: 351-6.

**[10]** Sulke N, Sugihara C, Hong P et al. The benefit of a remotely monitored implantable loop recorder as a first line investigation in unexplained syncope: the EaSyAS II trial. Europace 2016; 18(6): 912-8.

**[11]** Brignole M,Sutton R, Menozzi C et al. Early application of an implantable loop recorder allows effective specific therapy in patients with recurrent suspected neurally mediated syncope (ISSUE-2). European Heart Journal (2006) 27, 1085–1092

**[12]** https://www.biobanking.com/preventice-solutions-bodyguardian-mini-remote-cardiac-monitor-selected-for-uk-biobank-study (accessed 25 Sep 2022)

**[13]** Zio Service for detecting cardiac arrhythmias. NICE Guidance and guidelines MIB101; https://www.nice.org.uk/advice/mib101/chapter/The-technology (accessed 25 Sep 2022)

**[14]** NICE medical technology evaluation DHT pilot: Zio XT for detecting cardiac arrhythmias [GID-MT534]: https://www.nice.org.uk/guidance/indevelopment/gid-mt534 (accessed 25 Sep 2022)

**[15]** Hall R, John S, Monkhouse C et al. A patient centred evaluation of innovative

For peer review only

ambulatory ECG monitoring. Heart 2019; 105(Suppl 6): A37; 10.1136/heartjnl-2019-BCS.41

**[16]** Rosenberg MA1, Samuel M, Thosani A, Zimetbaum PJ. Use of a noninvasive continuous monitoring device in the management of atrial fibrillation: a pilot study. *Pacing Clin Electrophysiol.* 2013; 36(3): 328-33.

**[17]** Tung CE, Turakhia MP, Lansberg MG. Diagnostic Yield of Extended Cardiac Patch Monitoring in Patients with Stroke or TIA. Front. Neurol. 2014; 5:266.

**[18]** Summers A, Reed MJ. An evaluation of patient ownership and use and acceptability of smartphone technology within the emergency department. Eur J Emerg Med 2018; 25 (3): 224–225.

**[19]** M Solbiati, G Casazza, F Dipaola et al. Syncope recurrence and mortality a systematic review. Europace 2015; 17: 300–308.

**[20]** Edvardsson N, Frykman V, van Mechelen R et al. Use of an implantable loop recorder to increase the diagnostic yield in unexplained syncope: results from the PICTURE registry. Europace 2011; 13: 262–269

For peer review only

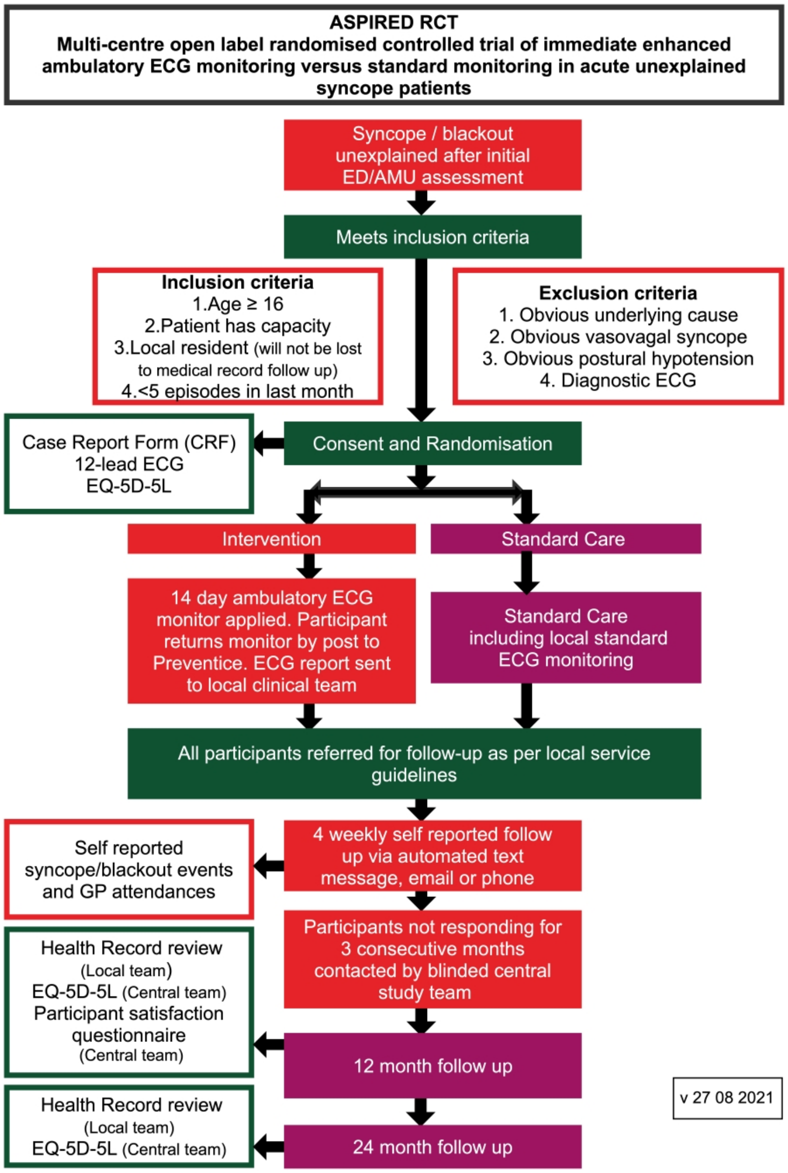


Figure 1: Schematic diagram of the study design and Participant Recruitment

1764x2486mm (72 x 72 DPI)

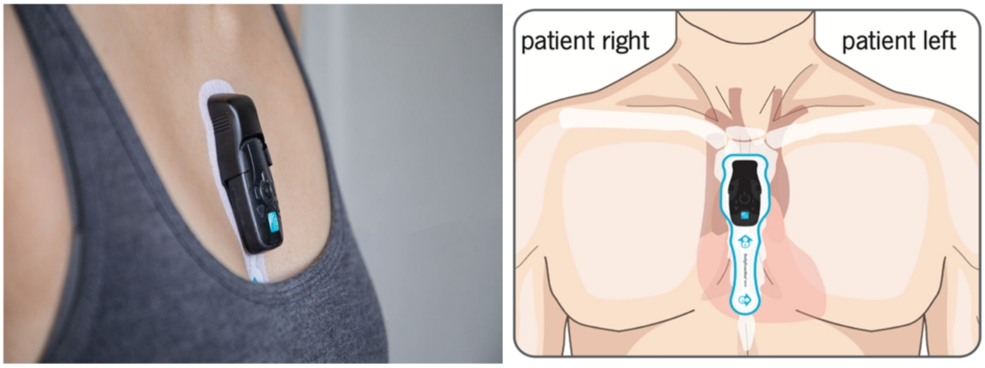


Figure 2: 14-day ambulatory heart monitor (Preventice BodyGuardian Mini)

473x177mm (144 x 144 DPI)

STANDARD PROTOCOL ITEMS: RECOMMENDATIONS FOR INTERVENTIONAL TRIALS

SPIRIT 2013 Checklist: Recommended items to address in a clinical trial protocol and related documents*

| Section/item | Item No | Description | Addressed on page number |
|---|---|---|---|
| **Administrative information** | | | |
| Title | 1 | Descriptive title identifying the study design, population, interventions, and, if applicable, trial acronym | ____1________ |
| Trial registration | 2a | Trial identifier and registry name. If not yet registered, name of intended registry | ____2________ |
| | 2b | All items from the World Health Organization Trial Registration Data Set | ____2________ |
| Protocol version | 3 | Date and version identifier | ____2________ |
| Funding | 4 | Sources and types of financial, material, and other support | ____12_______ |
| Roles and responsibilities | 5a | Names, affiliations, and roles of protocol contributors | ____1________ |
| | 5b | Name and contact information for the trial sponsor | ____1________ |
| | 5c | Role of study sponsor and funders, if any, in study design; collection, management, analysis, and interpretation of data; writing of the report; and the decision to submit the report for publication, including whether they will have ultimate authority over any of these activities | ___12________ |
| | 5d | Composition, roles, and responsibilities of the coordinating centre, steering committee, endpoint adjudication committee, data management team, and other individuals or groups overseeing the trial, if applicable (see Item 21a for data monitoring committee) | ___11________ |

For peer review only

**Introduction**

| | | | |
|---|---|---|---|
| Background and rationale | 6a | Description of research question and justification for undertaking the trial, including summary of relevant studies (published and unpublished) examining benefits and harms for each intervention | ____3________ |
| | 6b | Explanation for choice of comparators | ____3-4______ |
| Objectives | 7 | Specific objectives or hypotheses | _____4_______ |
| Trial design | 8 | Description of trial design including type of trial (eg, parallel group, crossover, factorial, single group), allocation ratio, and framework (eg, superiority, equivalence, noninferiority, exploratory) | ___5-9_______ |

**Methods: Participants, interventions, and outcomes**

| | | | |
|---|---|---|---|
| Study setting | 9 | Description of study settings (eg, community clinic, academic hospital) and list of countries where data will be collected. Reference to where list of study sites can be obtained | ____5________ |
| Eligibility criteria | 10 | Inclusion and exclusion criteria for participants. If applicable, eligibility criteria for study centres and individuals who will perform the interventions (eg, surgeons, psychotherapists) | __5-6________ |
| Interventions | 11a | Interventions for each group with sufficient detail to allow replication, including how and when they will be administered | ___6-9_______ |
| | 11b | Criteria for discontinuing or modifying allocated interventions for a given trial participant (eg, drug dose change in response to harms, participant request, or improving/worsening disease) | ___6-9_______ |
| | 11c | Strategies to improve adherence to intervention protocols, and any procedures for monitoring adherence (eg, drug tablet return, laboratory tests) | ___6-9_______ |
| | 11d | Relevant concomitant care and interventions that are permitted or prohibited during the trial | ___6-9_______ |
| Outcomes | 12 | Primary, secondary, and other outcomes, including the specific measurement variable (eg, systolic blood pressure), analysis metric (eg, change from baseline, final value, time to event), method of aggregation (eg, median, proportion), and time point for each outcome. Explanation of the clinical relevance of chosen efficacy and harm outcomes is strongly recommended | ___6-7_______ |
| Participant timeline | 13 | Time schedule of enrolment, interventions (including any run-ins and washouts), assessments, and visits for participants. A schematic diagram is highly recommended (see Figure) | __Table 4__ |



For peer review only - http://bmjopen.bmj.com/site/about/guidelines.xhtml

| | | | |
|---|---|---|---|
| Sample size | 14 | Estimated number of participants needed to achieve study objectives and how it was determined, including clinical and statistical assumptions supporting any sample size calculations | ___8-9_______ |
| Recruitment | 15 | Strategies for achieving adequate participant enrolment to reach target sample size | ___9_________ |

**Methods: Assignment of interventions (for controlled trials)**

Allocation:

| | | | |
|---|---|---|---|
| Sequence generation | 16a | Method of generating the allocation sequence (eg, computer-generated random numbers), and list of any factors for stratification. To reduce predictability of a random sequence, details of any planned restriction (eg, blocking) should be provided in a separate document that is unavailable to those who enrol participants or assign interventions | ____6________ |
| Allocation concealment mechanism | 16b | Mechanism of implementing the allocation sequence (eg, central telephone; sequentially numbered, opaque, sealed envelopes), describing any steps to conceal the sequence until interventions are assigned | ____6________ |
| Implementation | 16c | Who will generate the allocation sequence, who will enrol participants, and who will assign participants to interventions | ___6_________ |
| Blinding (masking) | 17a | Who will be blinded after assignment to interventions (eg, trial participants, care providers, outcome assessors, data analysts), and how | ____8________ |
| | 17b | If blinded, circumstances under which unblinding is permissible, and procedure for revealing a participant's allocated intervention during the trial | ____8________ |

**Methods: Data collection, management, and analysis**

| | | | |
|---|---|---|---|
| Data collection methods | 18a | Plans for assessment and collection of outcome, baseline, and other trial data, including any related processes to promote data quality (eg, duplicate measurements, training of assessors) and a description of study instruments (eg, questionnaires, laboratory tests) along with their reliability and validity, if known. Reference to where data collection forms can be found, if not in the protocol | __7-8________ |
| | 18b | Plans to promote participant retention and complete follow-up, including list of any outcome data to be collected for participants who discontinue or deviate from intervention protocols | __10_________ |



For peer review only - http://bmjopen.bmj.com/site/about/guidelines.xhtml

| | | | |
|---|---|---|---|
| Data management | 19 | Plans for data entry, coding, security, and storage, including any related processes to promote data quality (eg, double data entry; range checks for data values). Reference to where details of data management procedures can be found, if not in the protocol | ___11________ |
| Statistical methods | 20a | Statistical methods for analysing primary and secondary outcomes. Reference to where other details of the statistical analysis plan can be found, if not in the protocol | ___10________ |
| | 20b | Methods for any additional analyses (eg, subgroup and adjusted analyses) | ___10__________ |
| | 20c | Definition of analysis population relating to protocol non-adherence (eg, as randomised analysis), and any statistical methods to handle missing data (eg, multiple imputation) | ___10__________ |
| **Methods: Monitoring** | | | |
| Data monitoring | 21a | Composition of data monitoring committee (DMC); summary of its role and reporting structure; statement of whether it is independent from the sponsor and competing interests; and reference to where further details about its charter can be found, if not in the protocol. Alternatively, an explanation of why a DMC is not needed | __11_________ |
| | 21b | Description of any interim analyses and stopping guidelines, including who will have access to these interim results and make the final decision to terminate the trial | _11__________ |
| Harms | 22 | Plans for collecting, assessing, reporting, and managing solicited and spontaneously reported adverse events and other unintended effects of trial interventions or trial conduct | _11__________ |
| Auditing | 23 | Frequency and procedures for auditing trial conduct, if any, and whether the process will be independent from investigators and the sponsor | ___10-11______ |
| **Ethics and dissemination** | | | |
| Research ethics approval | 24 | Plans for seeking research ethics committee/institutional review board (REC/IRB) approval | ___2, 10______ |
| Protocol amendments | 25 | Plans for communicating important protocol modifications (eg, changes to eligibility criteria, outcomes, analyses) to relevant parties (eg, investigators, REC/IRBs, trial participants, trial registries, journals, regulators) | ___9-11_______ |

| | | | |
|---|---|---|---|
| Consent or assent | 26a | Who will obtain informed consent or assent from potential trial participants or authorised surrogates, and how (see Item 32) | __10_________ |
| | 26b | Additional consent provisions for collection and use of participant data and biological specimens in ancillary studies, if applicable | __10_________ |
| Confidentiality | 27 | How personal information about potential and enrolled participants will be collected, shared, and maintained in order to protect confidentiality before, during, and after the trial | __11_________ |
| Declaration of interests | 28 | Financial and other competing interests for principal investigators for the overall trial and each study site | __12_________ |
| Access to data | 29 | Statement of who will have access to the final trial dataset, and disclosure of contractual agreements that limit such access for investigators | __12_________ |
| Ancillary and post-trial care | 30 | Provisions, if any, for ancillary and post-trial care, and for compensation to those who suffer harm from trial participation | __11_________ |
| Dissemination policy | 31a | Plans for investigators and sponsor to communicate trial results to participants, healthcare professionals, the public, and other relevant groups (eg, via publication, reporting in results databases, or other data sharing arrangements), including any publication restrictions | _11___________ |
| | 31b | Authorship eligibility guidelines and any intended use of professional writers | ____11_________ |
| | 31c | Plans, if any, for granting public access to the full protocol, participant-level dataset, and statistical code | __12_________ |
| **Appendices** | | | |
| Informed consent materials | 32 | Model consent form and other related documentation given to participants and authorised surrogates | ___not included__ |
| Biological specimens | 33 | Plans for collection, laboratory evaluation, and storage of biological specimens for genetic or molecular analysis in the current trial and for future use in ancillary studies, if applicable | __n/a__________ |

*It is strongly recommended that this checklist be read in conjunction with the SPIRIT 2013 Explanation & Elaboration for important clarification on the items. Amendments to the protocol should be tracked and dated. The SPIRIT checklist is copyrighted by the SPIRIT Group under the Creative Commons "Attribution-NonCommercial-NoDerivs 3.0 Unported" license.



For peer review only - http://bmjopen.bmj.com/site/about/guidelines.xhtml

BMJ Open

# Multi-centre open label randomised controlled trial of immediate enhanced ambulatory ECG monitoring versus standard monitoring in acute unexplained syncope patients: The ASPIRED study.

| | |
|---|---|
| Journal: | *BMJ Open* |
| Manuscript ID | bmjopen-2022-069530.R1 |
| Article Type: | Protocol |
| Date Submitted by the Author: | 10-Jan-2023 |
| Complete List of Authors: | Reed, Matthew; The University of Edinburgh Usher Institute of Population Health Sciences and Informatics, Emergency Medicine Research Group Edinburgh (EMERGE)<br>Grubb, Neil; Edinburgh Royal Infirmary, Department of Cardiology<br>Lang, Chris; Edinburgh Royal Infirmary, Department of Cardiology<br>Goodacre, Steve; The University of Sheffield, Medical Care Research Unit<br>O'Brien, Rachel; Edinburgh Royal Infirmary, Emergency Medicine Research Group Edinburgh (EMERGE)<br>Weir, Christopher; The University of Edinburgh Usher Institute of Population Health Sciences and Informatics, Edinburgh Clinical Trials Unit, Usher Institute<br>Thokala, Praveen; The University of Sheffield<br>Freeman, Nicola; Edinburgh Royal Infirmary, Emergency Medicine Research Group Edinburgh (EMERGE)<br>Blackstock, Caroline; Edinburgh Royal Infirmary, Emergency Medicine Research Group Edinburgh (EMERGE)<br>Dinsmore, Lynn; The University of Edinburgh Usher Institute of Population Health Sciences and Informatics, Edinburgh Clinical Trials Unit, Usher Institute<br>Boyd, Julia; The University of Edinburgh Usher Institute of Population Health Sciences and Informatics, Edinburgh Clinical Trials Unit<br>Adamestam, Imad; The University of Edinburgh Usher Institute of Population Health Sciences and Informatics, Edinburgh Clinical Trials Unit, Usher Institute<br>Macrae, Pam; ASPIRED study Patient Advisory Group<br>Hannigan, Robert; ASPIRED study Patient Advisory Group<br>Lobban, Trudie; Arrhythmia Alliance |
| <b>Primary Subject Heading</b>: | Emergency medicine |
| Secondary Subject Heading: | Cardiovascular medicine, Health economics |
| Keywords: | ACCIDENT & EMERGENCY MEDICINE, Adult cardiology < CARDIOLOGY, Pacing & electrophysiology < CARDIOLOGY, GENERAL MEDICINE (see Internal Medicine), Cardiology < INTERNAL MEDICINE |
| | |

SCHOLARONE™
Manuscripts

**Title:** Multi-centre open label randomised controlled trial of immediate enhanced ambulatory ECG monitoring versus standard monitoring in acute unexplained syncope patients: The ASPIRED study.

**Authors:** Matthew J Reed [1,2], Neil Grubb [3], Chris Lang [3], Steve Goodacre [4], Rachel O'Brien [1], Christopher Weir [5], Praveen Thokala [4], Nicola Freeman [1], Caroline Blackstock [1], Lynn Dinsmore [5], Julia Boyd [5], Imad Adamestam [5], Pam Macrae [6], Robert Hannigan [6], Trudie Lobban [6]

1. Emergency Medicine Research Group Edinburgh (EMERGE), Royal Infirmary of Edinburgh, Edinburgh, UK
2. Acute Care Edinburgh, Usher Institute, University of Edinburgh, Edinburgh, UK
3. Department of Cardiology, Royal Infirmary of Edinburgh, Edinburgh, UK
4. School of Health and Related Research (ScHARR), University of Sheffield
5. Edinburgh Clinical Trials Unit (ECTU), University of Edinburgh, Edinburgh, UK
6. ASPIRED study Patient Advisory Group

**Corresponding Author**
Professor Matthew J Reed, Emergency Medicine Research Group Edinburgh (EMERGE), Department of Emergency Medicine, Royal Infirmary of Edinburgh, Edinburgh, UK.

Email: mattreed@ed.ac.uk/ v1mreed3@ed.ac.uk
Tel: +44 (0) 131 242 1284

**Trial Sponsor:** The University of Edinburgh and Lothian Health Board, ACCORD, The Queen's Medical Research Institute, 47 Little France Crescent, Edinburgh, EH16 4TJ, UK.

**Word Count:** 4417 (4,000) words, 20 references, 2 Figures, 5 Tables

**Abstract Word Count:** 198

For peer review only

**ABSTRACT:**

**Introduction:** Diagnosing underlying arrhythmia in Emergency Department (ED) syncope patients is difficult. There is evidence that diagnostic yield for detecting underlying arrhythmia is highest when cardiac monitoring devices are applied early, ideally at the index visit **[1-3].** This strategy has the potential to change current syncope management from low diagnostic yield Holter to higher yield ambulatory monitoring, reduce episodes of syncope, reduce risk of recurrence and its potential serious consequences, reduce hospital admissions, reduce overall health costs and increase quality of life by allowing earlier diagnosis, treatment and exclusion of clinically important arrhythmias.

**Methods and analyses:** This is a UK open prospective parallel group multicentre randomised controlled trial of an immediate 14-day ambulatory patch heart monitor versus standard care in 2234 patients presenting acutely with unexplained syncope. Our patient focussed primary endpoint will be number of episodes of syncope at one year. Health economic evaluation will estimate the incremental cost per syncope episode avoided and quality-adjusted life year gained.

**Ethics and dissemination:** Informed consent for participation will be sought. The ASPIRED trial received a favourable ethical opinion from South East Scotland Research Ethics Committee 01 (21/SS/0073). Results will be disseminated via scientific publication, lay summary, and visual abstract.

**Registration Details:** ISRCTN 10278811 - https://doi.org/10.1186/ISRCTN10278811
Protocol version 3; 14 March 2022

**ARTICLE SUMMARY**

**Strengths & Limitations of this study**

- The ASPIRED trial uses non-blinded randomisation to compare immediate enhanced ambulatory ECG monitoring versus standard monitoring in acute unexplained syncope patients.
- The primary outcome measure is a patient focussed measure very relevant to the population of patients who suffer syncope.
- We are using health economic analysis and a separate embedded qualitative study to assess the wider implications of using immediate enhanced ambulatory ECG monitoring in this population.
- In contrast to the few published studies on ambulatory monitoring in syncope patients which are generally single centred, without any comparative group and with heterogeneous patient populations and device capabilities, this study is multicentred and includes a standard care group.

**Key Words:** Ambulatory Electrocardiography Monitoring, Cardiac Arrhythmias, and Syncope, Diagnostic Tests

For peer review only

# INTRODUCTION

## Background and Rationale

Syncope (or blackout) is common; 650,000 patients present to UK EDs every year. The 3 underlying causes are neurocardiogenic (including simple faint), postural hypotension (blood pressure fall on standing) and cardiac disease (structural heart disease or cardiac arrhythmia). Diagnosis is difficult and is not made in around 50% of patients after assessment **[1-4]**. Whilst vasovagal and postural syncope are relatively benign, serious pathology (affecting 7 out of every 100 patients at one month after ED attendance **[5]**) include arrhythmia (an abnormal heart rhythm). When cardiac arrhythmias are detected, they are most commonly asystolic pauses, reflex bradycardia or advanced atrioventricular block, with tachycardia being the minority **[6].**

The difficulty in the ED is to differentiate between the causes of syncope and identify patients at higher risk. This can be complicated as many patients have fully recovered on ED arrival and their examination and presenting electrocardiogram (ECG) may both be normal. The current method for establishing cardiac arrhythmia as the cause of syncope rests on correlating the arrhythmia with symptoms. The lack of efficacy and availability of commonly used monitoring devices means most high and medium risk patients are admitted to hospital for observation and telemetry (if available), with escalating costs **[1]**. Unfortunately, many (around 50% of patients after assessment) still end up being discharged without a diagnosis **[7]**.

In general, syncope reoccurs in around 50% of patients within a year. Recurring episodes increase hospital admissions, health costs and importantly reduce the quality of life of patients. Whilst there is a wide variation in the literature with respect to the number of syncope episodes and recurrence rates pre and post treatment **[6, 8-10]** once a cardiac arrhythmia diagnosis is made and treatment initiated, around only 10% of patients will have a 1-year recurrence **[11]** and syncope episodes will drop by over 90% **[11]**.

There is evidence that the diagnostic yield for detecting underlying arrhythmia is highest when cardiac monitoring devices are applied early after syncope, ideally at the index visit **[1-3]**. To solve the problems with current routine ECG monitoring devices, several novel ambulatory devices have recently been developed **[1, 12-15]**. These devices are non-invasive, water-resistant, have no leads or wires, are discreet to wear and are CE-marked for clinical use in the UK. They continuously monitor the heart for up to 14 days including during sleep, in the shower, and during moderate exercise and some have a button for patients to capture symptomatic events. They offer medium duration high fidelity ECG recording and are well tolerated **[16,17]**. At the end of the monitoring period, patients return the devices back to the company (by mail) or NHS (to be downloaded) for analysis. The PATCH-ED pilot study helped establish trial methods and numbers of available participants informing this RCT **[1]**. This pilot study showed a novel ECG monitoring device was able to detect serious cardiac arrhythmia

For peer review only

requiring treatment in a significant proportion of patients and demonstrated the potential to influence clinical management decisions relating to hospital admission and participant outcomes.

The ASPIRED randomised controlled trial (RCT) will compare a novel ambulatory cardiac monitoring device with standard practice in syncope patients. We hypothesise that applying cardiac monitoring early after syncope at the index visit is the optimum strategy to detect, diagnose, treat, and exclude underlying cardiac arrhythmia.

**Study Objectives:**

**Primary:** To determine whether immediate, enhanced (14-day) ambulatory ECG monitoring decreases the number of self-reported episodes of syncope at one year compared to standard care monitoring in acute unexplained syncope patients.

**Secondary:** To determine whether immediate, enhanced (14-day) ambulatory ECG monitoring in acute unexplained syncope patients can:

1. Decrease the number of episodes of self-reported syncope at 90 days, and 2 years compared to standard care monitoring.
2. Decrease the time to detection of clinically significant cardiac arrhythmia compared to standard care monitoring.
3. Increase the rate of detection of clinically significant cardiac arrhythmia at 90 days and 1 year compared to standard care monitoring.
4. Increase the rate of ECG/symptom correlation at 90 days and 1 year compared to standard care monitoring.
5. Demonstrate cost effectiveness compared to standard care monitoring.
6. Decrease the number of episodes of syncope identified in the medical records at 90 days, 1 and 2 years compared to standard care monitoring.
7. Decrease the index hospital admission rate and duration of hospital stay compared to standard care monitoring
8. Decrease 90 days, 1- and 2-year syncope recurrence rates (identified in the medical records and self-reported) compared to standard care monitoring
9. Increase patient satisfaction compared to standard care monitoring
10. Decrease the rate of 30 day, 1 and 2 year all cause death compared to standard care monitoring
11. In the intervention group, by reporting the timing of detection of clinically significant cardiac arrhythmia what is the optimum duration of acute ambulatory ECG monitoring
12. Increase the affect rate of diagnostic testing and therapeutic intervention.

For peer review only

## METHODS AND ANALYSIS

### Study design

This is a UK open prospective parallel group multicentre RCT of an immediate 14-day ambulatory patch heart monitor versus standard care monitoring in 2234 participants presenting acutely with unexplained syncope. The patient focussed primary endpoint will be number of episodes of syncope at one year **[Table 1]**.

**Table 1:** Study summary in PICO format

| | |
|---|---|
| **P: Population** | Adults presenting acutely to UK hospitals with syncope remaining unexplained after initial ED/AMU assessment |
| **I: Intervention** | 14-day ambulatory heart monitor placed on patients |
| **C: Comparator** | Standard care monitoring |
| **O: Primary Outcome** | Number of self-reported episodes of syncope at one year |

**Study Eligibility:** 2234 adult (16 years or older) participants presenting acutely to UK hospitals with syncope remaining unexplained after initial ED or Acute Medicine Unit (AMU) assessment will be enrolled. Syncope will be defined as transient loss of consciousness (TLOC) with inability to maintain postural tone and immediate complete spontaneous recovery without medical intervention **[18]**.

**Inclusion Criteria:**

1. Syncope remains unexplained after initial ED/AMU assessment.
2. Aged ≥ 16 years
3. Patient has capacity
4. Local resident (i.e. resident within local health board so will not be lost to medical record follow up)
5. <5 self-reported episodes of syncope in the previous month

**Exclusion Criteria:**

1. Obvious underlying cause after assessment
   a. Features of vasovagal syncope **(see Table 2) AND** absence of structural heart disease **AND** normal physical examination **AND** normal ECG
   b. Arrhythmia on pre-hospital or hospital ECG as likely cause of syncope
   c. Postural hypotension (symptomatic postural drop >20 mmHg **AND** suggestive history)
   d. Confirmed diagnosis of Pulmonary Embolus or Acute Myocardial Infarction
   e. Radiological diagnosis or clinical signs/symptoms of cerebrovascular accident/transient ischemic attack or subarachnoid haemorrhage
   f. Evidence of:
      i. Haemorrhage
      ii. Alcohol or illicit drugs

For peer review only - http://bmjopen.bmj.com/site/about/guidelines.xhtml

iii. Epileptic seizure
iv. Hypoglycemia
v. Head trauma
vi. Other obvious cause of syncope as presumptive cause of TLoC

2. Inability to consent
3. Previous recruitment into the study
4. Patient in custody or prison
5. Aged <16 years
6. Patient does not reside within local health board and will therefore be lost to medical record follow up
7. 5 or more self-reported episodes of syncope in previous 4 weeks

Pregnancy is not an exclusion criteria.

**Table 2:** Features of vasovagal / postural syncope

| |
|---|
| Associated with typical symptoms of reflex syncope (e.g. light-headedness, feeling of warmth, nausea, vomiting) |
| After sudden unexpected unpleasant sight, sound, smell, or pain |
| In association with micturition, defaecation, cough, laughter, venepuncture, blood phobia |
| After prolonged standing or crowded, hot places |
| During a meal or after eating a meal |
| With head rotation or pressure on carotid sinus (e.g. tumour, shaving, tight collars) |
| Associated with standing up quickly from a sitting or lying position |
| Long history (years) of recurrent syncope with low-risk features with the same characteristics of the current episode |

**Participant Recruitment:** Recruitment will take place in around 30-40 NHS acute tertiary and district hospitals. **Figure 1** summarises the study design and participant recruitment.

**Randomisation and Interventions**

Randomisation will be performed using a web-based randomisation service to ensure allocation concealment, managed by Edinburgh Clinical Trials Unit (ECTU). The allocation sequence will be created by an ECTU database programmer using computer-generated pseudo-random numbers. Participants will be randomised, 1:1, between the two study arms. Stratification by site will be used to ensure there will not be a significant imbalance between the number randomised to intervention and control at any site. Stratification by other site-level characteristics will not be performed.

Standard care will include all care usually given to unexplained syncope patients at each participating site along with some form of standard care monitoring such as but not limited

For peer review only

to wired inpatient telemetry, Holter style monitoring or implantable ECG recorder. The study will be conducted over 4 years. Recruitment will take place over 18 months. Intervention group participants will be fitted with a 14-day ambulatory heart monitor. All participants will be followed-up for 2 years after index event.

**Outcome Measures:**

**Primary Endpoint:** Number of self-reported episodes of syncope at one year.

**Secondary Endpoints:**

1. Within trial cost effectiveness (cost per syncope avoided and cost per quality adjusted life year [QALY] gained), and lifetime cost per QALY gained at (a) 1 year and (b) 2 years
2. Number of self-reported episodes of syncope at (a) 90 days and (b) 2 years, those identified in the medical records at (c) 90 days, (d) 1 year and (e) 2 years, and syncope recurrence rate at (f) 90 days, (g) 1 year and (h) 2 years
3. Index presentation hospital (a) admission rate and (b) duration of hospital stay
4. Patient Satisfaction (measured using a patient questionnaire) at 1 year
5. Clinically significant cardiac arrhythmia (Serious and/or symptomatic cardiac arrhythmia **Table 3**) at (a) 90-days, (b) 1 year and (c) 2 years.
6. (a) 30 day, (b) 1 year and (c) 2 year all cause death
7. Detection of diagnostic ECG/symptom correlation (symptomatic) at (a) 90-days, (b) 1 year and (c) 2 years.
8. Time to detect clinically significant cardiac arrhythmia (i.e. time to clinician being aware)
9. In the intervention group, duration of enhanced ambulatory ECG monitoring required to detect clinically significant cardiac arrhythmia
10. Number and type of diagnostic tests and therapeutic interventions at (a) 1 year and (b) 2 years

**Table 3:** Definitions of clinically significant cardiac arrhythmias

| |
|---|
| Ventricular Fibrillation (VF) * |
| Ventricular Tachycardia (VT) ≥120 beats per minute (bpm) for ≥30 seconds * |
| VT ≥120 beats per minute for <30 seconds (≥4 beats) * |
| Complete or 3rd degree heart block * |
| Second degree atrioventricular heart block Mobitz type II * |
| Second degree atrioventricular heart block Mobitz type I |
| Pause ≥6 seconds * |
| Sinus pause ≥2.5 seconds when awake or ≥4 seconds at night (but <6 seconds) |
| Sinus bradycardia <30 beats/minute * |
| Bradycardia <40 beats per minute for ≥30 seconds * |
| Bradycardia <40 beats per minute for <30 seconds |
| Sick sinus syndrome with alternating sinus bradycardia and tachycardia |

For peer review only

| |
|---|
| Junctional / idioventricular rhythm ≥30 seconds in duration |
| Supraventricular tachycardia >100 beats per minute ≥30 seconds in duration |
| Atrial flutter/fibrillation with ventricular rate >100 bpm or <60 bpm ≥30 seconds in |
| New Atrial flutter/fibrillation ≥30 seconds in duration |

* Defined as 'Serious' clinically significant cardiac arrhythmia

Nb. All arrhythmias will also be classed as symptomatic or asymptomatic during monitoring period

**Trial assessments**

**Participant identification:** Potential participants will be identified from EDs/AMUs or other acute settings and approached either during their ED/AMU or hospital stay or contacted after discharge and invited to take part in the study.

**Participant consent:** Potential participants will be given a Participant Information Sheet (PIS). Potential participants will be randomised within 72 hours of their hospital attendance. If a patient has been discharged from hospital, then they will be contacted by the local clinical team or research team (if part of the clinical team), and a copy of the PIS will be emailed or posted out to the patient. The PIS will also be available on the trial website. A delegated member of the local study team will seek verbal consent over the telephone and will sign the consent form on behalf of the participant. The original consent form will be filed in the Investigator Site File (ISF) and a copy will be sent to the participant. Alternatively, the patient can attend the hospital with travel expenses, to provide written informed consent in person. The ambulatory ECG device can be sent to the participant's address or collected from the local study team if randomised to the intervention arm. Each participating centre will upload screening information of non-identifiable potentially eligible patients who were approached to participate in the study, onto the study database. Participants are free to withdraw from the study at any point or can be withdrawn by the Investigator

For peer review only

Participants will have an eCRF completed at randomisation **[Table 4]**. Recruited participants regardless of allocation group, will be referred for syncope assessment as per local service protocol and subsequent investigations will be arranged at the discretion of the treating clinician. Participants randomised to the intervention arm will be fitted with a 14-day ambulatory heart monitor (Preventice BodyGuardian Mini) applied by the study team as soon after randomisation as possible. The ambulatory ECG monitor will be placed on the participant's chest wall over the sternum, connected directly to an ECG electrode sticker **[Figure 2]**. The participant's skin does not require shaving but is cleaned prior to attaching the device, which are easily removed by the participant after 14 days. The monitor can be worn by both women and men.

**Table 4: Study Assessments**

| Assessment | Screening | Day 1 baseline | Monthly | 90 days | Every 3 months | 1 year | 2 years |
|---|---|---|---|---|---|---|---|
| **Window of time for evaluation** | n/a | n/a | +/- 14 days | +/- 14 days | n/a | +/- 30 days | +/- 30 days |
| Assessment of Eligibility Criteria | X | | | | | | |
| Informed consent | X | | | | | | |
| eCRF completion including demographic data and contact details | X | | | | | | |
| Routine clinical care (e.g. ECG) | X | | | | | | |
| Randomisation | | X | | | | | |
| Intervention group participants fitted with a 14-day ambulatory heart monitor | | X | | | | | |
| Referral for syncope assessment and standard care monitoring as per local service protocol to be seen ideally within 4-6 weeks of the index event especially if discharged from ED or if this did not occur during index admission. | | X | | | | | |
| EQ-5D-5L questionnaires | | X | | | | X | X |
| NHS resource utilisation data from routine hospital electronic healthcare records extracted by the local study team) | | | | X | | X | X |
| Participant contacted on a 4-weekly basis via automated text message, email or phone with a link to a brief web-based questionnaire asking for the number of syncope events experienced since last response, and the number of GP attendances for any reason. | | | X | | | | |
| Participants not responding for 3 consecutive months to receive phone call from central study team to collect missing data, ensure no syncope episodes have occurred and to encourage continued future engagement. Participants with a mean of 5 or more episodes/month will also receive a phone call from the central study team to ensure that participants are recording true syncope events and are seeking appropriate medical advice. | | | | | X | | |
| Participant satisfaction questionnaire | | | | | | X | |

Participants will be required to press the button on the heart monitor after any syncopal event. They will also record symptomatic episodes in a paper diary. The participant will wear

For peer review only

the ambulatory ECG monitor for a maximum of 14 days after which they will remove and return it in a pre-paid envelope to Preventice UK. The monitor will be reported by a Cardiac Physiologist, shared with the participant's local study team, placed in the participant's health record, and shared with the participant's treating clinician. Treatment of device findings will be at the discretion of the treating clinician at each site. The participant's GP will be informed that the participant has been enrolled in the study and will be informed of the results of any ECG investigations via routine hospital clinical correspondence. Participants will be informed of the results of any ECG investigations via routine hospital clinical correspondence. Any study participant with a serious arrhythmia **[Table 3]** on the ECG report will be contacted as soon as possible by the local team and managed appropriately according to local policy.

All participants will be contacted on a 4-weekly basis +/- 14 days for 2 years via automated text message or email (whichever they prefer) with a link to a brief web-based questionnaire asking for the number of syncope events experienced since their last response and how many of these they attended hospital for. Those who are unable to access digital forms of communication will receive phone calls. They will also be asked since their last response how many times they have visited their GP practice for any reason including all face-to-face, telephone and online consultations. This will import directly into the central ECTU study database. This patient reported data will be used to inform the primary endpoint. The small number of participants unable to access digital forms of communication and participants not responding for 3 consecutive months will receive a phone call from the central study team (blinded to participant's study arm allocation) to collect missing data, ensure no syncope episodes have occurred and to encourage continued future engagement. Participants with a mean of 5 or more episodes/month will also receive a phone call from the central study team to ensure that participants are recording true syncope events and are seeking appropriate medical advice.

We were very thoughtful about the potential impact of Covid-19 when designing this study including ensuring ambulatory ECG monitors can be sent directly to patients and designing participant follow-up to ensure no additional research related hospital visits are required.

Participants will also be contacted at one and two years +/- 30 days, by the central study team (blinded to participants study arm allocation) to complete a quality-of-life questionnaire. In the event of non-response, participants will be contacted on up to 3 occasions. The participants' involvement in the study will cease at 2 years. Endpoint data including NHS resource utilisation will be extracted by the local study team from routine hospital electronic healthcare records at 90 days, 1 and 2 years and will be entered into a bespoke database. ECTU will collect and clean primary data and perform primary and secondary analyses.

For peer review only

**Timelines:** The study started on 1st August 2021 and the first patient was recruited on 15th July 2022. Recruitment is planned to end on 31st December 2023, the with study completing on 31st July 2025.

**Sample Size:**
2234 participants (1117 standard and 1117 intervention) presenting acutely with syncope whose syncope remains unexplained after initial ED/AMU assessment will be recruited. Using an estimated mean 1-year recurrence rate in untreated patients of 42.5% **[6]** and a reduction in 1-year recurrence rate to 10% in patients who are treated **[11,19]** then the hypothesis is a 1-year recurrence rate in the standard group (2% diagnosed with a symptomatic significant arrhythmia with 90% of these expected to not have recurrence after treatment) of 40.7% compared to a recurrence rate of 33.1% in the treatment group (10.5% of pilot participants diagnosed with symptomatic significant arrhythmia at 90 days with 90% of these expected to not have recurrence after cardiac arrhythmia treatment) equating to an event rate ratio of 0.81. A more conservative effect size of 6% (40% vs 34%) will be assumed corresponding to a more conservative event rate ratio of 0.85. A large study of unexplained untreated syncope patients **[20]** suggests a median (IQR) number of events in the preceding 2 years of 3 (2-4) with a median (IQR) per year of 2 (1-3.5). The ESC Guidelines **[6]** suggest that the number of events has a good fit to a Poisson distribution and the untreated event rate post-attendance is about 70% reduced from the pre-attendance rate. The ESC guidelines **[6]** and PICTURE **[20]** suggest a mean number of events per participant of approximately 1 during 1 year of follow-up. If we assume that this follows a negative binomial distribution (which allows for "over dispersion" versus the Poisson distribution) then a study of 1064 participants per group would have 90% power (two-sided significance level=5%, over dispersion=0.25) to detect an event rate ratio of 0.85.

The study will recruit an extra 5% in each arm (i.e. 1117 participants per arm; 2234 in total) to allow for drop-out/loss to follow up although we expect this to be low (<1% in pilot) and drop out due to death (<1% in pilot). It is expected that most people will respond to some text/email follow-ups, but few will respond to all. We will therefore call any participant who has not responded for 3 consecutive months to ensure no syncope episodes have occurred and to encourage continued future engagement. Participants will be defined as lost to follow up only if both one-year electronic patient health record data and one-year self-reported data is unavailable.

**Study Progression Criteria:** This study will include an internal recruitment pilot phase with stop-go recruitment milestone criteria to mitigate risk to the funder. This internal pilot will be used to confirm recruitment rates and aims to recruit the first 400 participants (almost one fifth of the sample size) from 10 sites by the end of study month 13. By the end of study month 13, the aim is to have 400 participants (18%) enrolled with an average recruitment

For peer review only

rate/site/month of at least 5 participants in the best 60% of sites, with at least 10 sites open **[Table 5]**.

**Table 5:** Internal recruitment pilot study progression criteria

| By end of study month 13 | Red | Amber | Green |
|---|---|---|---|
| Total number of participants recruited | ≤200 | 201-399 | ≥400 |
| % recruitment of total required | 9% | 10-17% | 18% |
| Average recruitment rate/site/active months in the best 60% of sites * | 3 | 4 | 5 |
| Number of sites open | <5 | 5-10 | >10 |

* Sites recruitment rate will be calculated from site opening date.

If by the end of study month 19, overall recruitment is less than 1300 participants (58%), OR average recruitment rate/site/month is less than 6 participants in the best 60% of sites, OR there are less than 20 sites recruiting, we will further expand the number of NHS sites recruiting. We will also consider whether study extension is required.

**Data Analysis Plan:** The primary outcome, number of self-reported episodes of syncope in the 12 months following randomisation, will be analysed by negative binomial regression. The primary outcome event rate ratio (14-day ambulatory heart monitor vs standard care) will be reported with its 95% confidence interval. An offset term for follow-up duration will be included to account for participants with partial follow up.

The secondary outcomes for the number of syncope episodes at 90 days and 2 years, will be analysed similarly. Binary secondary outcomes will be analysed by logistic regression, reporting the odds ratio (14-day ambulatory heart monitor vs standard care) and its 95% confidence interval. Full details of analysis, including the estimand(s) of interest and methods for handling missing data, will be written into a Statistical Analysis Plan, which will be finalised prior to database lock without knowledge of the unblinded treatment allocations.

**Cost Effectiveness Analysis:** Anonymised electronic healthcare record data will be sent to our health economist in Sheffield to apply unit costs and tariffs, to estimate within trial costs and QALYs, and then to undertake lifetime economic modelling. Both within trial and lifetime cost-effectiveness analysis will be performed. In within trial analysis, costs will be estimated by applying national unit costs to items of resource use (monitoring, hospitalisation, treatment, health, and social care) to estimate the mean cost per participant in each arm of the trial. Cost-effectiveness will then be estimated as the incremental cost per syncope episode avoided and the incremental cost per quality-adjusted life year (QALY) gained, with QALYs being estimated from EQ5D questionnaires. Lifetime cost-effectiveness will be estimated using decision analytic modelling from published sources of life expectancy, annual costs and corresponding annual utilities. This will explore the potential impact of events, such as

For peer review only

syncope episode resulting in death or injury, that have consequences beyond the timeframe of the trial.

**Interim analysis:** In addition to the blinded sample size review to ensure that the trial achieves the required statistical power, there will be a single interim futility analysis for the primary outcome performed after the 18th month of recruitment (end of study month 25). At this point 6 months of 1 year follow-up data will be available. We anticipate at least 400 participants will have undergone 12 months follow-up for the primary outcome at this point and will be able to be analysed in this futility analysis.

**Patient and Public Involvement (PPI):** The ASPIRED study PPI group is made up of patient representatives, lay members and a representative from the Arrhythmia Alliance. They have been involved in informing the study research questions and study protocol in particularly the methods and timings of patient follow-up, and the development of all patient facing information.

## ETHICS AND DISSEMINATION

The ASPIRED trial received a favourable ethical opinion from South East Scotland Research Ethics Committee 01 (21/SS/0073).

**Participant Capacity and Consent:** Capacity will be assessed by the research team or a clinician responsible for the treatment of the participant. The trial excludes patients who have inability to give informed consent and therefore patients with temporary incapacity due to their current illness or with permanent incapacity will not be recruited.

**Safety Considerations:**

**Bias:** The primary outcome is a quantitative endpoint (number of syncope episodes) collected through automated participant reporting importing directly into the ECTU central study database to reduce reporting bias. Central research staff who phone participants will be blinded to participant allocation.

**Adverse events:** A secondary endpoint for the study is serious outcomes at 90-days, 1 and 2 years. This data will therefore be routinely collected as part of the study and not recorded as an Adverse Event (AE). Hospital admission will also not be recorded as an AE. The only AEs recorded will be those directly related to the use of initial ambulatory ECG recording both in the intervention and standard care groups. Participants will be asked through the automated monthly email/text questionnaire at month 2, whether they suffered any complications related to wearing any monitoring devices in the first 2 months of the trial.

A Trial Steering Committee (TSC) will be established to oversee the conduct and progress of the trial. The details of the TSC will be captured in a separate charter. An independent Data

For peer review only

Monitoring Committee (DMC) will be established to oversee the safety of participants in the trial. The details of the DMC will be captured in a separate charter.

**Data Management:** Identifiable data collected by the study will not be transferred to any external individuals or organisations outside of the sponsoring organisations.

**Study Dissemination:** We will disseminate the results of this study widely through high impact peer-reviewed publications, presentations at international conferences, local and national websites, charity newsletters and websites and media outlets such as television and radio. We will also share our results through specific interest groups such as Arrhythmia alliance and disseminate findings amongst guideline development groups such as ESC, SIGN, NICE and American College of Cardiology (ACC).

**Registration Details**:

Registered at ISRCTN (10278811 https://doi.org/10.1186/ISRCTN10278811)

**AUTHOR CONTRIBUTIONS**: MJR, NG, CL, SG, ROB, CW, PT, NF, CB, LD, JB, IA, PM, RH and TL all contributed to the conception and design of the work including writing of the study protocol, drafting the work, and revising it critically for important intellectual content, final approval of the version to be published, and agree to be accountable for all aspects of the work in ensuring that questions related to the accuracy or integrity of any part of the work are appropriately investigated and resolved. MJR is responsible for the overall content as guarantor.

**ACKNOWLEDGEMENTS:** We thank all patients, lay members, and members of patient and public involvement groups who gave feedback to improve the study design.

**FUNDING STATEMENT:** This work was supported by the British Heart Foundation (BHF) grant number CS/F/20/190016. The funder and sponsor have reviewed the study design and protocol but have no role in the collection, management, analysis, and interpretation of data; writing of the report or the decision to submit the paper for publication. MJR is supported by an NHS Research Scotland Career Researcher Clinician award. All other authors declared no competing interests.

**COMPETING INTERESTS STATEMENT:** MJR is supported by an NHS Research Scotland Career Researcher Clinician award. All other authors declared no competing interests.

**DATA STATEMENT:** All study data and metadata will be available upon reasonable request to ECTUdatashare@ed.ac.uk in accordance with the ASPIRED Study Data Sharing Plan version 1.0; 9th January 2023.

**FIGURE and TABLE LEGENDS**

**Figure 1:** Schematic diagram of the study design and Participant Recruitment

For peer review only

**Figure 2:** 14-day ambulatory heart monitor (Preventice BodyGuardian Mini)

For peer review only

**REFERENCES**

**[1]** Reed MJ, Grubb NJ, Lang CC, Gray AJ, Simpson K, MaCraild AJ, Weir CJ. Diagnostic yield of an ambulatory patch monitor in patients with unexplained syncope after initial evaluation in the Emergency Department: The PATCH-ED study. *Emerg Med J* 2018; 35:477–485.

**[2]** Locati ET, Moya A, Oliveira M, et al. External prolonged electrocardiogram monitoring in unexplained syncope and palpitations: results of the SYNARR-Flash study. Europace 2016; 18: 1265–1272

**[3]** Thiruganasambandamoorthy V, Rowe BH, Sivilotti MLA, et al. Duration of Electrocardiographic Monitoring of Emergency Department Patients With Syncope. Circulation 2019; 139: 1396–1406

**[4]** Reed MJ, Newby DE, Coull AJ et al. Risk stratification Of Syncope in the Emergency department: The ROSE study. *J American College Cardiology* 2010;55(8): 713-721.

**[5]** Reed MJ, Henderson SS, Newby DE, et al. One-year prognosis after syncope and the failure of the ROSE decision instrument to predict 1-year adverse events. *Ann Emerg Med* 2011; 58(3): 250-6.

**[6]** Brignole M, Reed MJ et al. 2018 ESC Guidelines for the diagnosis and management of syncope. *European Heart Journal* 2018; 39(21): 1883-1948; doi:10.1093/eurheartj/ehy037

**[7]** Tattersall L, Reed MJ. The Inpatient Management of Syncope. Emerg Med J. 2010; 27: 870-872.

**[8]** Solbiati M, Costantino G, Casazza G et al. Implantable loop recorder versus conventional diagnostic workup for unexplained recurrent syncope. Cochrane Database Syst Rev.2016; doi: 10.1002/14651858.CD011637.pub2.

**[9]** Farwell DJ, Freemantle N, Sulke N. The clinical impact of implantable loop recorders in patients with syncope. European Heart Journal 2006; 27: 351-6.

**[10]** Sulke N, Sugihara C, Hong P et al. The benefit of a remotely monitored implantable loop recorder as a first line investigation in unexplained syncope: the EaSyAS II trial. Europace 2016; 18(6): 912-8.

**[11]** Brignole M,Sutton R, Menozzi C et al. Early application of an implantable loop recorder allows effective specific therapy in patients with recurrent suspected neurally mediated syncope (ISSUE-2). European Heart Journal (2006) 27, 1085–1092

**[12]** https://www.biobanking.com/preventice-solutions-bodyguardian-mini-remote-cardiac-monitor-selected-for-uk-biobank-study (accessed 25 Sep 2022)

**[13]** Zio Service for detecting cardiac arrhythmias. NICE Guidance and guidelines MIB101; https://www.nice.org.uk/advice/mib101/chapter/The-technology (accessed 25 Sep 2022)

**[14]** NICE medical technology evaluation DHT pilot: Zio XT for detecting cardiac arrhythmias [GID-MT534]: https://www.nice.org.uk/guidance/indevelopment/gid-mt534 (accessed 25 Sep 2022)

**[15]** Hall R, John S, Monkhouse C et al. A patient centred evaluation of innovative

For peer review only

ambulatory ECG monitoring. Heart 2019; 105(Suppl 6): A37; 10.1136/heartjnl-2019-BCS.41

**[16]** Rosenberg MA1, Samuel M, Thosani A, Zimetbaum PJ. Use of a noninvasive continuous monitoring device in the management of atrial fibrillation: a pilot study. *Pacing Clin Electrophysiol.* 2013; 36(3): 328-33.

**[17]** Tung CE, Turakhia MP, Lansberg MG. Diagnostic Yield of Extended Cardiac Patch Monitoring in Patients with Stroke or TIA. Front. Neurol. 2014; 5:266.

**[18]** Summers A, Reed MJ. An evaluation of patient ownership and use and acceptability of smartphone technology within the emergency department. Eur J Emerg Med 2018; 25 (3): 224–225.

**[19]** M Solbiati, G Casazza, F Dipaola et al. Syncope recurrence and mortality a systematic review. Europace 2015; 17: 300–308.

**[20]** Edvardsson N, Frykman V, van Mechelen R et al. Use of an implantable loop recorder to increase the diagnostic yield in unexplained syncope: results from the PICTURE registry. Europace 2011; 13: 262–269

For peer review only

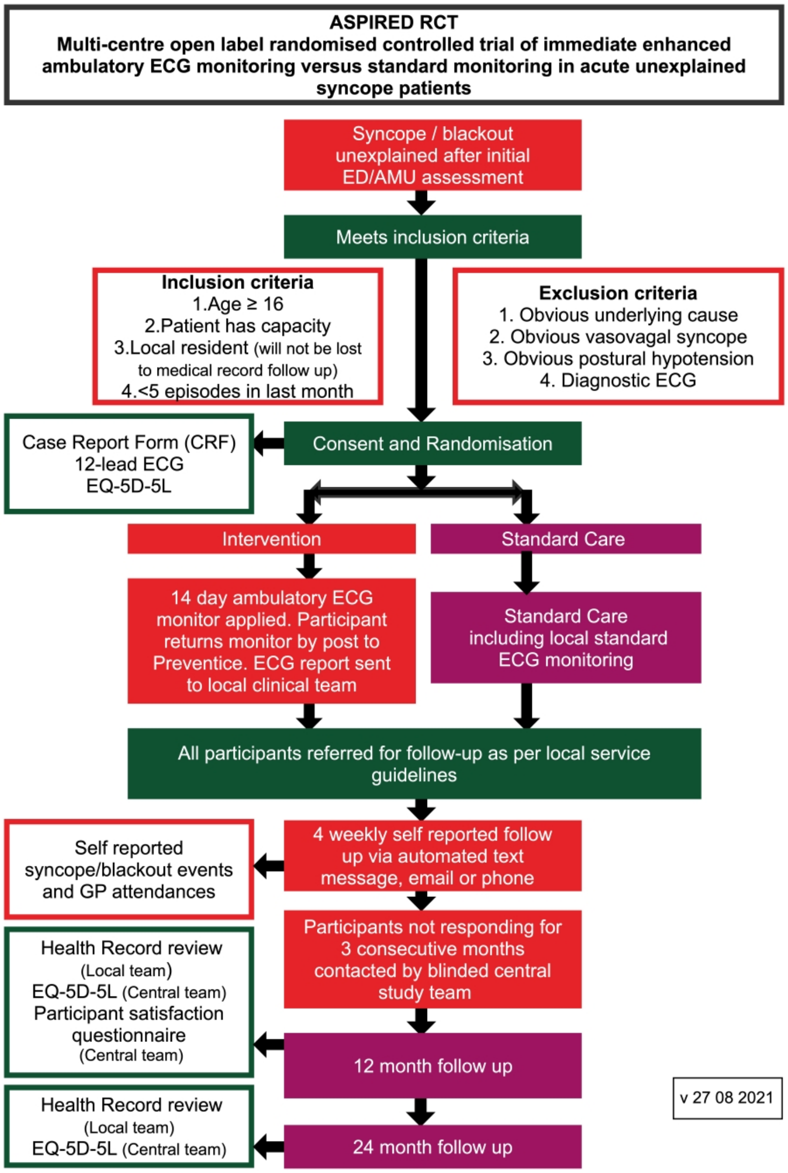


Figure 1: Schematic diagram of the study design and Participant Recruitment

1764x2486mm (72 x 72 DPI)

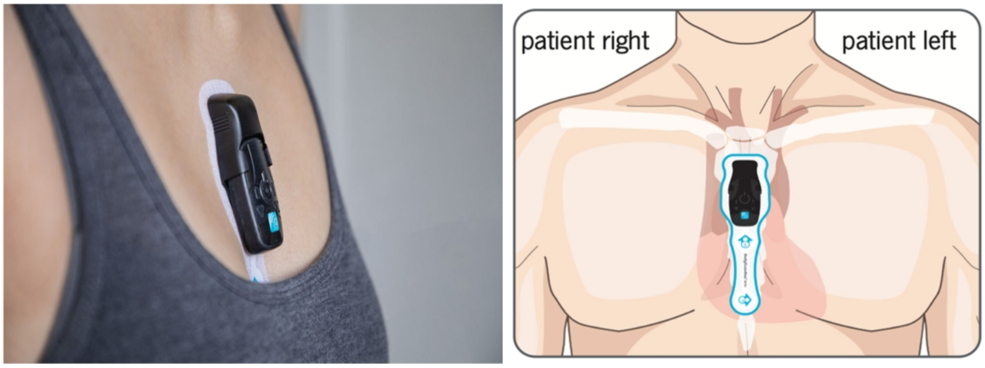


Figure 2: 14-day ambulatory heart monitor (Preventice BodyGuardian Mini)

473x177mm (144 x 144 DPI)
