# Supplementary material for: Multicentre open label randomised controlled trial of immediate enhanced ambulatory ECG monitoring versus standard monitoring in acute unexplained syncope patients: the ASPIRED study
Source: BMJ Open. 2023 Feb 23;13(2):e069530. doi: 10.1136/bmjopen-2022-069530 (PMC9950891; doi:10.1136/bmjopen-2022-069530)
Supplement: Reviewer comments [file bmjopen-2022-069530.reviewer_comments.pdf]

PEER REVIEW HISTORY

BMJ Open publishes all reviews undertaken for accepted manuscripts. Reviewers are asked to complete a checklist review form (**http://bmjopen.bmj.com/site/about/resources/checklist.pdf**) and are provided with free text boxes to elaborate on their assessment. These free text comments are reproduced below.

ARTICLE DETAILS

**TITLE (PROVISIONAL)**	Multi-centre open label randomised controlled trial of immediate enhanced ambulatory ECG monitoring versus standard monitoring in acute unexplained syncope patients: The ASPIRED study.
**AUTHORS**	Reed, Matthew; Grubb, Neil; Lang, Chris; Goodacre, Steve; O'Brien, Rachel; Weir, Christopher; Thokala, Praveen; Freeman, Nicola; Blackstock, Caroline; Dinsmore, Lynn; Boyd, Julia; Adamestam, Imad; Macrae, Pam; Hannigan, Robert; Lobban, Trudie

This article was not externally reviewed at BMJ Open. Protocols that have been independently assessed prior to submission to BMJ Open are usually fast-tracked to publication on the grounds that further substantial changes will not be possible. This independent assessment will usually be external, independent review for both a substantial grant award from a non-commercial or government funder and ethics approval.
